# PHP-Family Diesterase from *Novosphingobium* with Broad Specificity and High Catalytic Efficiency against Organophosphate Flame-Retardant Derived Diesters

**DOI:** 10.1021/acs.biochem.4c00350

**Published:** 2024-12-02

**Authors:** Preston Garner, Andrew C. Davis, Andrew N. Bigley

**Affiliations:** Department of Chemistry and Physics, Southwestern Oklahoma State University, Weatherford, Oklahoma 73096, United States

## Abstract

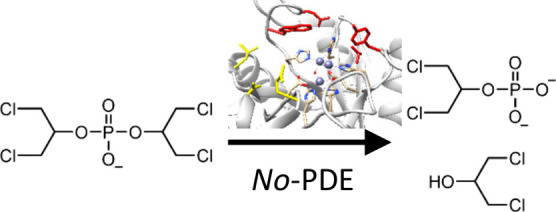

Organophosphate flame retardants have been widely used in plastic products since the early 2000s. Unfortunately, these compounds leach out of the plastics over time and are carcinogenic, developmental toxins, and endocrine disruptors. Due to the high usage levels and stable nature of the compounds, widespread contamination of the environment has now been observed. Despite their recent introduction into the environment, bacteria from the *Sphingomonadaceae* family have evolved a three-step hydrolytic pathway to utilize these compounds. The second step in this pathway in *Sphingobium* sp. TCM1 is catalyzed by *Sb*-PDE, which is a member of the polymerase and histidinol phosphatase (PHP) family of phosphatases. This enzyme is only the second case of a PHP-family enzyme capable of hydrolyzing phosphodiesters. Bioinformatics analysis has now been used to identify a second PHP diesterase from *Novosphingobium* sp. EMRT-2 (*No*-PDE). Kinetic characterization of *Sb*-PDE and *No*-PDE with authentic organophosphate flame-retardant diesters demonstrates that these enzymes are true diesterases with more than 1000-fold selectivity for the diesterase activity seen in some cases. Synthesis of a wide array of authentic flame-retardant diesters has allowed the substrate specificity of these enzymes to be determined, and mutagenic analysis of the active site residues has identified key residues that give rise to the high levels of diesterase activity. Despite high sequence identity, *No*-PDE is found to have a broader substrate specificity against flame-retardant derived diesters, and *k*_cat_/*K*_m_ values greater than 10^4^ M^–1^ s^–1^ are seen with the best substrates.

Flame retardants have been required in plastics and durable foam products since the 1970s to limit the flammability of these ubiquitous goods.^1^ Since the discontinuation of the polybrominated diphenyl ethers in the early 2000s, the use of organophosphate flame retardants has been rising, with hundreds of tons currently produced each year.^2^ The organophosphate flame retardants are phosphotriesters. Unlike the better-known organophosphate neurotoxins, used primarily as insecticides, organophosphate flame retardants are extremely stable compounds. While not neurotoxic, the organophosphate flame retardants such as triphenyl phosphate, tris-2-chloroethyl phosphate, tris-1,3-dichloroisopropyl phosphate, tris-2,3-dibromopropyl phosphate, and tris-2-butoxyethyl phosphate are carcinogens, developmental toxins, and endocrine disruptors.^2^ The high usage rates and long environmental persistence have led to widespread contamination of the environment including water, air, sediments, and, particularly concerning for children, household dust.^3^

Despite their recent introduction into the environment, bacteria from several lineages have evolved catabolic pathways to utilize the phosphate in the flame retardants.^4^ The pathway which evolved in several members of the *Sphingomonadaceae* family proceeds via the sequential hydrolytic cleavage of the three ester groups by a phosphotriestease (*Sb*-PTE), a phosphodiesterase (*Sb*-PDE), and a phosphatase (*Sb*-PhoK;^5−7^Figure 1A). Organophosphate flame retardants are not substrates for typical phosphotriesterase enzymes due to a lack of an activated leaving group; however, in *Sphingomonadaceae* the evolved phosphotriesterase readily hydrolyzes the first ester group of the organophosphate flame retardants.^8,9^ A great deal of work has been done to understand the evolution of these phosphotriesterases.^10,11^ By contrast, very little is known about the diesterases that catalyze the second step in the pathway. The identity of the diesterase involved in the degradation of the organophosphate flame retardants is only known in the strain *Sphingobium* sp. TCM1 (*Sb*-PDE). Sequence analysis identified *Sb*-PDE as a member of the Polymerase and Histidinol Phosphatase family (PHP). Members of the PHP family are well-known as phosphatases and as structural units in DNA polymerases.^12,13^ Tests of *Sb*-PDE with the model compounds bis-*p*-nitrophenyl phosphate (**1**) and *p*-nitrophenyl phosphate (**11**) demonstrated a clear selectivity for the diesterase activity over the phosphatase reaction,^5^ but a lack of a commercial source for flame-retardant-derived diesters prevented the determination of the substrate specificity for *Sb*-PDE.

**Figure 1:**
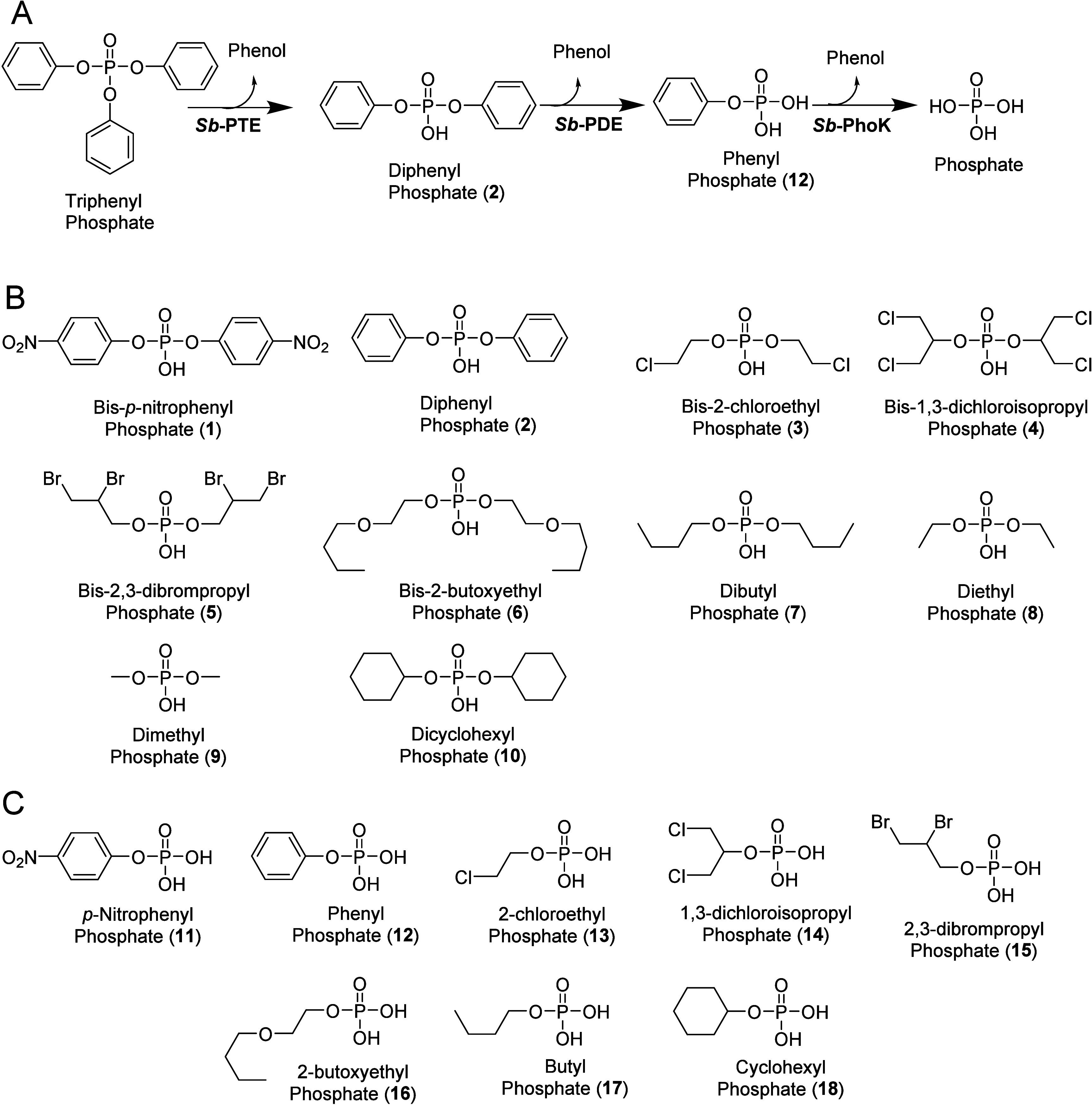
(A) Metabolic pathway for triphenyl phosphate degradation in *Sphingobium* sp. TCM1. (B) Structures of diesters derived from common organophosphate flame-retardants, industrial compounds and insecticides. (C) Corresponding monoesters to diesters shown in part B.

To determine if the diesterase activity seen in *Sb-*PDE might be present in additional members of the PHP family, the protein sequence of *Sb*-PDE was used as a search sequence on the Enzyme Function Initiative–Enzyme Similarity Tool to construct a sequence similarity network from the 623 homologues identified with a minimal evolutionary relationship defined by an alignment score of 40 (∼30% identity). Aside from *Sb*-PDE, none of the identified homologues have a known function, and only one has a known structure (PDB: 3E38). *Sb*-PDE appears in a small isolated cluster of seven proteins (PDE-cluster; Figure 2). Five of the identified proteins were found to be identical in sequence to *Sb*-PDE, while one sequence from *Sphingobium indicum* (*Si-*PDE) was 83% identical, and a third from *Novosphingobium* sp. EMRT-2 (*No*-PDE) was found to be 97% identical. Somewhat surprisingly, the maximal sequence identity to any other protein not in the PDE cluster was only 36%. Also surprising was that, while all of the proteins in the PDE cluster were from the *Sphingomonadaceae* family, only one other sequence in the 623 identified homologues was from a *Sphingomonadaceae* species.

**Figure 2:**
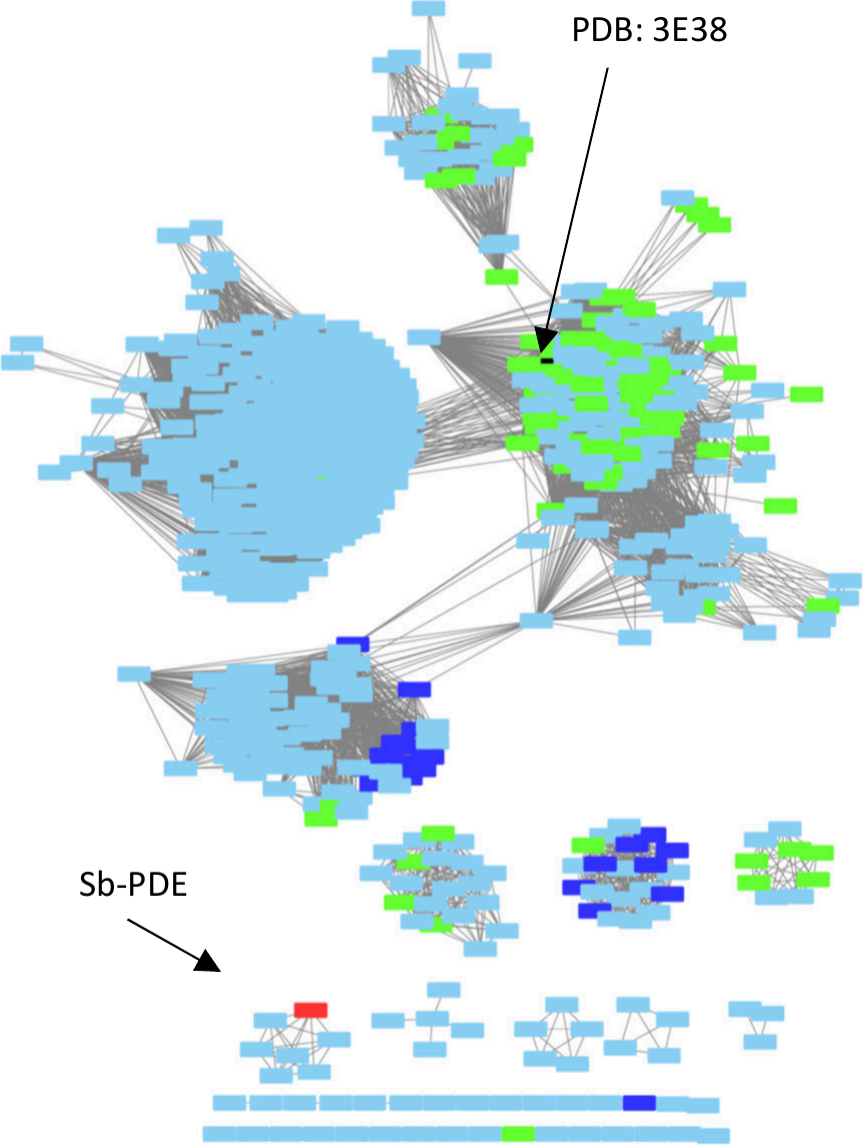
Sequence similarity network of Sb-PDE and evolutionarily related members of the PHP family of enzymes. Each rectangle represents a unique sequence from the Uniprot database, while each line represents an evolutionary relationship (alignment score) of 85 or greater. *Sb*-PDE is colored red and labeled. The single protein with a known structure (PDB: 3E38) is colored black and labeled. Proteins annotated as histidinol phosphatases are shown in green. Proteins annotated as phosphotransferases are colored dark blue. The remaining (light blue) proteins are annotated as the PHP-domain or PolIII-domain proteins.

The genes for *Sb*-PDE, *Si*-PDE, and *No*-PDE were obtained as synthetic constructs. Despite multiple attempts, no expression of *Si*-PDE was observed. *No*-PDE and *Sb*-PDE were successfully expressed and purified to homogeneity with yields of 5–10 mg/L of culture. Metal analysis indicates that the enzymes were fully constituted with a trinuclear zinc site (Table S1).

To allow the determination of the substrate profile for *Sb*-PDE and *No*-PDE, a set of diesters was synthesized to complement commercially available compounds including the model compound bis-p-nitrophenyl phosphate (**1**), the flame-retardant derived diesters, diphenyl phosphate (**2**), bis-2-chlorolethyl phosphate (**3**), bis-1,3-dichloroisopropyl phosphate (**4**), bis-2,3-dibromopropyl phosphate (**5**), bis-2-butoxyethyl phosphate (**6**), the industrial phosphate diesters dibutyl phosphate (**7**) and dicyclohexyl phosphate (**10**), and the insecticide derived diesters diethyl phosphate (**8**) and dimethyl phosphate (**9**). Standard Michelis-Menton kinetics were performed using compounds **1** and **2** using UV/vis spectroscopy (Figure S1 and Table S2). For the remainder of the compounds, ^31^P NMR was utilized to follow total hydrolysis reactions which yields only *k*_cat_/*K*_m_ (Figures S2–S7).

Against diphenyl phosphate (**2**), both enzymes have high *k*_cat_ values (49 s^–1^) and high enzymatic efficiency (>10^4^ M^–1^ s^–1^), but *Sb*-PDE demonstrated 2.4-fold better *k*_cat_/*K*_m_ (5.0 × 10^4^ M^–1^ s^–1^ vs 2.4 × 10^4^ M^–1^ s^–1^; Table 1). The best substrate for *No*-PDE was the diphenyl phosphate (**2**), but only slightly reduced was bis-1,3-dichloroisopropyl phosphate (**4**; *k*_cat_/*K*_m_ = 1.3 × 10^4^ M^–1^ s^–1^), which was ∼5-fold better than *Sb*-PDE. The *No*-PDE activity for bis-2,3-dibromophenyl phosphate (**5**; *k*_cat_/*K*_m_ = 4.1 × 10^3^ M^–1^ s^–1^) and bis-2-butoxyethyl phosphate (**6**; *k*_cat_/*K*_m_ = 1.4 × 10^3^ M^–1^ s^–1^) was lower though still greater than 10^3^ M^–1^ s^–1^. The least efficient activity was seen with 2-chloroethyl phosphate (**2**; *k*_cat_/*K*_m_ = 7.7 × 10^2^ M^–1^ s^–1^), though that was 20-fold better than the activity seen with *Sb*-PDE (*k*_cat_/*K*_m_ = 3.9 × 10^1^ M^–1^ s^–1^). *No*-PDE and *Sb*-PDE showed no activity against diethyl phosphate (**8**) or dimethyl phosphate (**9**; Figure S8). *Sb*-PDE was slightly more efficient against dicyclohexyl phosphate (**10**; *k*_cat_/*K*_m_ = 8 × 10^2^ M^–1^ s^–1^ vs 6.4 × 10^2^ M^–1^ s^–1^), while *No*-PDE was 3.4-fold more efficient for dibutyl phosphate (**7**; *k*_cat_/*K*_m_ = 1.2 × 10^2^ M^–1^ s^–1^ vs 3.5 × 10^1^ M^–1^ s^–1^) than *Sb*-PDE

**Table 1:** Kinetic Rate Constants for *No*-PDE and *Sb*-PDE^a^

	*No*-PDE			*Sb*-PDE		
compound	*k*_cat_ (s^–1^)	*K*_m_ (mM)	*k*_cat_/*K*_m_ (M^–1^ s^–1^)	*k*_cat_ (s^–1^)	*K*_m_ (mM)	*k*_cat_/*K*_m_ (M^–1^ s^–1^)
diesters						
1	6.7	0.62	1.1 × 10^4^	19.8	0.14	1.4 × 10^5^
2	49	2.3	2.1 × 10^4^	49	0.9	5.0 × 10^4^
3	nd	nd	7.7 × 10^2^	nd	nd	3.9 × 10^1^
4	nd	nd	1.3 × 10^4^	nd	nd	2.8 × 10^3^
5	nd	nd	4.1 × 10^3^	nd	nd	3.4 × 10^3^
6	nd	nd	1.4 × 10^3^	nd	nd	7.0 × 10^2^
7	nd	nd	1.2 × 10^2^	nd	nd	3.5 × 10^1^
8	NO	NO	<1 × 10^0^	NO	NO	<1 × 10^0^
9	NO	NO	<1 × 10^0^	NO	NO	<1 × 10^0^
10	nd	nd	6.4 × 10^2^	nd	nd	8 × 10^2^
monoesters						
11	0.7	1.4	5.0 × 10^2^	0.63	0.14	4.4 × 10^3^
12	nd	nd	3.3 × 10^1^	0.21	1.4	1.6 × 10^2^
14	nd	nd	<1 × 10^1^	NO	NO	<1 × 10^0^
17	nd	nd	<5 × 10^0^	NO	NO	<1 × 10^0^

a nd = not determined. NO = reaction not observed. Experimental errors were generally less than 10% and are given in the SI.

To test the possibility that *No*-PDE and *Sb*-PDE were phosphatases with promiscuous diesterase activity, the enzymes were characterized with the phosphomonoesters *p*-nitrophenyl phosphate (**11**) and phenyl phosphate (**12**). Both enzymes demonstrated a strong preference for the diesterase reaction (Table 1). *No*-PDE prefers the diester by a factor of 22-fold for the *p*-nitrophenyl leaving group (1.1 × 10^4^ vs 5.0 × 10^2^ M^–1^ s^–1^) and 636-fold with the phenyl leaving group (2.1 × 10^4^ vs 3.3 × 10^1^ M^–1^ s^–1^). With *p*-nitrophenol, *Sb*-PDE was slightly more specific with a 32-fold preference for the diester (1.4 × 10^5^ vs 4.4 × 10^3^ M^–1^ s^–1^), but *Sb*-PDE was less specific with the phenyl leaving group showing a 312-fold preference (5 × 10^4^ vs 1.6 × 10^2^ M^–1^ s^–1^). Interestingly, both enzymes demonstrated substantially higher *k*_cat_ values for the unactivated phenyl leaving group in the diesters than the highly activated *p*-nitrophenyl leaving group.

The ^31^P NMR analysis used to analyze diester hydrolysis also allowed for the observation of phosphatase activity with the resulting monoesters (compounds **13**–**18)**. In all cases, the diesterase reaction proceeded to completion prior to the observation of any phosphate due to the phosphatase reaction (Figures S2–S7). Extended incubation (up to 72 h) with *No*-PDE did demonstrate phosphatase activity with 1,3-dichloroisopropyl phosphate (**14**) and butyl phosphate (**17**; Figure S9), but the level of activity was too low to determine kinetic parameters. None of the other compounds tested with *No*-PDE demonstrated phosphatase activity with the enzyme generated monoesters, nor did any of the compounds tested with *Sb*-PDE (Figure S10).

*Sb*-PDE is only the second known example of a PHP-family enzyme capable of hydrolyzing diesters.^5,14^ The other case is the PHP enzyme Elen0235 from *Eggerthella lenta*, which hydrolyzes both the phosphodiester and the resulting monoester bond in a cyclic phosphodiester substrate.^14,15^ By contrast, *Sb*-PDE and *No*-PDE are specific diesterases.^5^ In some cases, greater than 600-fold selectivity is seen for the diesterase reaction over a phosphatase reaction with the same leaving group. The structural mechanism of the evolution of diesterase activity in the PHP family remains unknown. The single homologue of known structure (PDB: 3E38), which is assumed to be a phosphatase, demonstrates the typical trinuclear metal center of the PHP family^16^ (Figure 3). *Sb*-PDE and *No*-PDE both have fully conserved complements of metal binding residues with the α-metal ligated by H61, H63, and D260. The β-metal is ligated by H203 and H142. Additionally, both the α- and β-metals are bridged by a water molecule and E135. The γ-metal is coordinated by residues D68, H93, and H262 (Figure S11).

**Figure 3:**
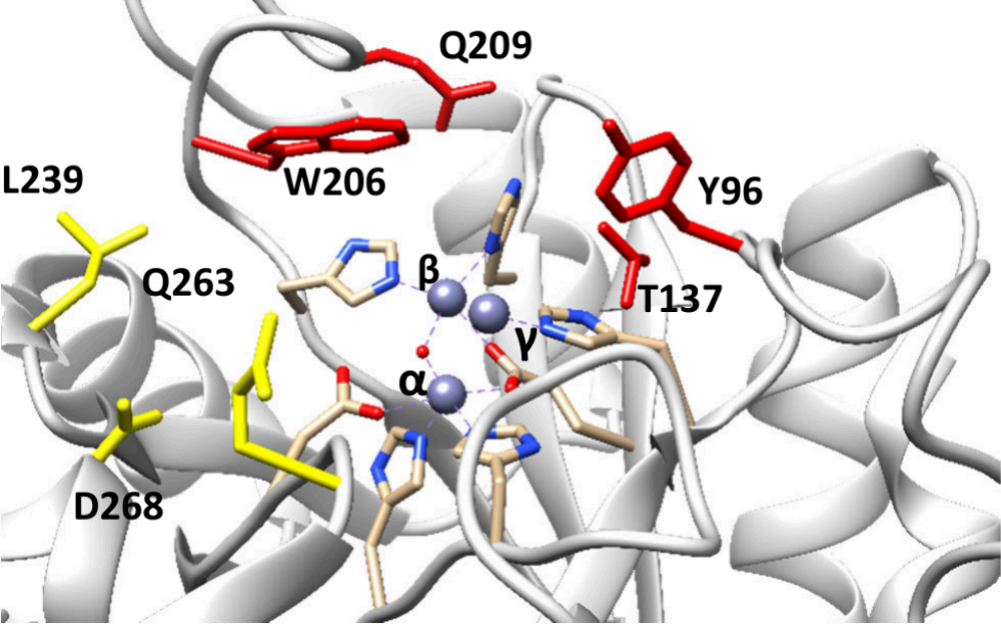
Active site of *No*-PDE homologue from *B. vulgatus* (PDB: 3e38) showing the trivalent metal center where the α- and β-metals activate the nucleophilic water and the γ-metal acts as a Lewis acid to the leaving group. Additional metal ligating residues are not shown for the sake of clarity. The pocket for the leaving group is lined by residues shown in red, while the side ester for a diester substrate would bind in the pocket lined by residues in yellow. Numbering from *No*-PDE.

The substrate binding pocket in PDB 3E38 consists of a cleft for the leaving group, which is lined by residues Y96, T137, W206, and Q206 (Figure S11). On the opposite side of the active site is a second depression to accommodate the side ester in a diester substrate. The side ester pocket is lined by residues L239, Q236, and D268. The substrate binding residues are not conserved, with only T137 being the same in *No*-PDE and PDB 3E38. To test the significance of these changes on the selectivity for the diesterase activity, a series of mutants of *No*-PDE was constructed, changing the substrate site residues to the corresponding residue in PDB 3E38 or alanine. Each variant was characterized using *p*-nitrophenyl di- and monoesters (compounds **1** and **11**) and phenol containing di- and monoesters (compounds **2** and **12**) to determine the effect on the selectivity for the diesterase reaction. Kinetic parameters for all variants are listed in Table S1.

Changes to three of the four leaving group pocket residues significantly altered the selectivity. The wild-type enzyme is more selective for the diesterase reaction with the authentic flame-retardant-derived diester diphenyl phosphate (**2**) compared to the model substrate bis-*p*-nitrophenyl phosphate (**1**; Figure 4). Mutation of I96 to the corresponding aromatic residue in PDB 3E38 results in a ∼10-fold shift toward the phosphatase reaction. I206W had the largest effect on the selectivity with the phenyl leaving group showing a 60-fold reduction in selectivity. This effect was not observed with the *p*-nitrophenyl leaving group, which showed only a 2-fold reduction in selectivity. Interestingly, the alanine mutants at these positions have a smaller effect on the selectivity with the phenyl leaving group. With the *p*-nitrophenyl leaving group, I96A increases the selectivity by 10-fold, while I206A diminished it 4-fold. Mutations at position 209 in the *No*-PDE leaving group pocket had nearly no effect on the selectivity. The final leaving group pocket residue in *No*-PDE is conserved in PDB 3E38, and the null mutant T137A had less than a 2-fold reduction in selectivity with the phenyl leaving group and increased the selectivity more than 4-fold with the *p*-nitrophenyl leaving group.

**Figure 4:**
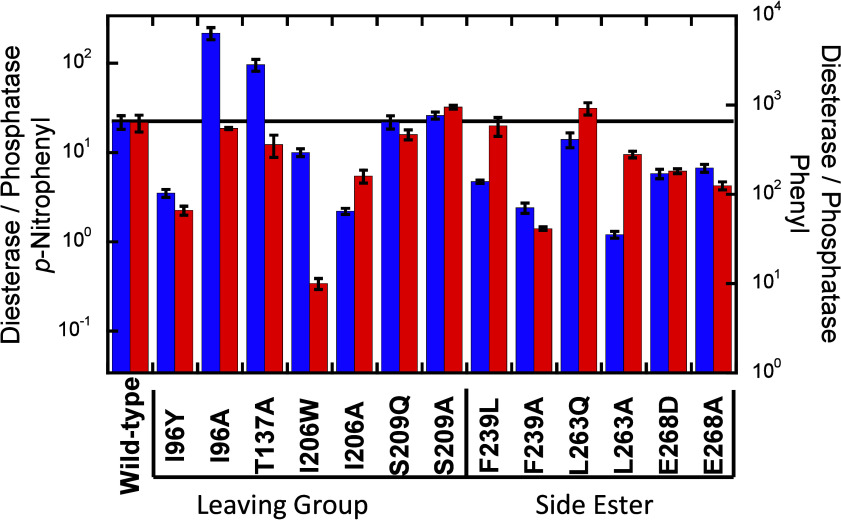
Ratio of diesterase to phosphatase activity for variants of *No*-PDE. Data for bis-*p*-nitrophenyl phosphate (**1**) and *p*-nitrophenyl phosphate (**11**) are shown in blue and correspond to the left-hand axis. Data for diphenyl phosphate (**2**) and phenyl phosphate (**12**) are shown in red and correspond to the right-hand axis.

Mutation of F239 and L263 in the proposed side ester pocket to their corresponding residues in PDB 3E38 resulted in less than a 2-fold change in the diesterase selectivity for the phenyl leaving group. However, the F239L mutation decreased the selectivity with the *p*-nitrophenyl leaving group by 5-fold. Mutation of the third ester pocket residue, E268, decreased the diesterase selectivity by ∼4-fold for both leaving groups. The side ester pocket residues do appear to be important for the diesterase activity as the alanine mutants at positions 239 and 263 show. The F239A mutation diminished the selectivity by more than 10-fold for both leaving groups, and the L263A mutation effectively eliminates selectivity with the *p*-nitrophenyl leaving group while only reducing the selectivity ∼2-fold with the phenyl leaving group.

This work has demonstrated that *Sb*-PDE and *No*-PDE are selective diesterases with broad specificity and high enzymatic efficiency for multiple flame-retardant derived diesters. Mutagenic analysis suggests that the novel diesterase activity in the PHP family has been brought about by extensive remodeling of the substrate binding site.

## Accession Codes

Sb-PDE:EMBL-CDS:BAX25588.1; No-PDE:EMBL-CDS:QCI95559.1; Si-PDE:EMBL-CDS:CCW15874.1; Elen0235:gi|257790010; 3E38-PHP:EMBL-CDS:ABR41129.1; and PDB: 3E38.
