# Supplementary material for: PHP-Family Diesterase from Novosphingobium with Broad Specificity and High Catalytic Efficiency against Organophosphate Flame-Retardant Derived Diesters
Source: Biochemistry. 2024 Dec 2;63(24):3189–93. doi: 10.1021/acs.biochem.4c00350 (PMC11656708; doi:10.1021/acs.biochem.4c00350)
Supplement: Supplementary file 1 — bi4c00350_si_001.pdf [file bi4c00350_si_001.pdf]

Supplementary Information

PHP-family diesterase from *Novosphingobium* with broad specificity and high catalytic efficiency against organophosphate flame-retardant derived diesters

Preston Garner, Andrew C. Davis, and Andrew N. Bigley*

Department of Chemistry and Physics, Southwestern Oklahoma State University, Weatherford, Oklahoma, 73096

Table S1: Flame AA analysis of Zn bound to proteins.

Variant	Protein / Zn ratio
No-PDE WT	2.7
Sb-PDE WT	2.9
No-PDE I96Y	2.3
No-PDE I96A	2.6
No-PDE T137A	3.0
No-PDE I206W	2.8
No-PDE I206A	3.0
No-PDE S209Q	3.0
No-PDE S209A	2.7
No-PDE F239L	2.9
No-PDE F239A	3.0
No-PDE L263Q	2.9
No-PDE L263A	3.0
No-PDE E268D	3.0
No-PDE E268 A	3.0

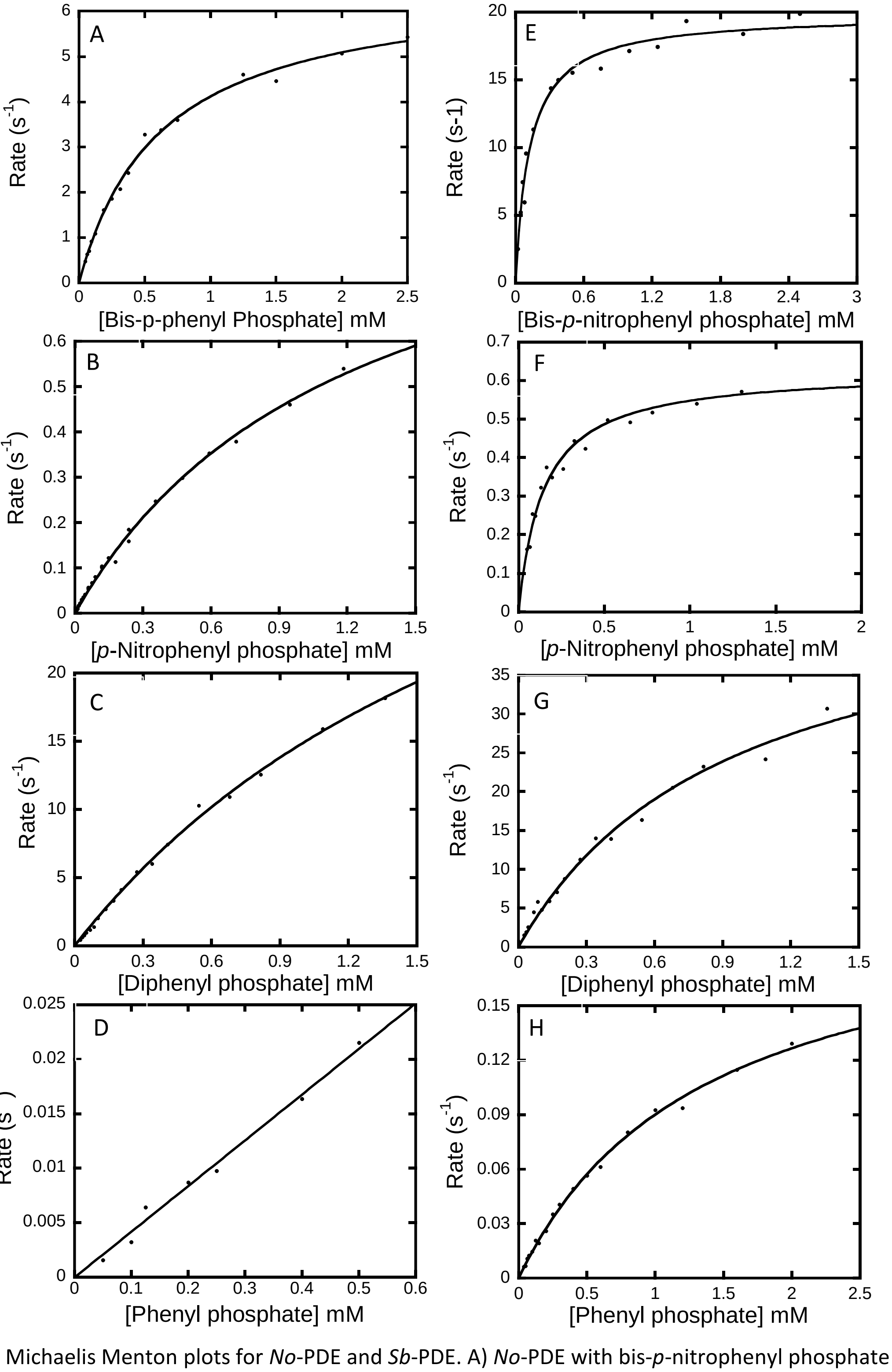

Figure S1: Michaelis Menton plots for *No*-PDE and *Sb*-PDE. A) *No*-PDE with bis-*p*-nitrophenyl phosphate (**1**). B) *No*-PDE with *p*-nitrophenyl phosphate (**11**). C) *No*-PDE with diphenyl phosphate (**2**). D) *No*-PDE with phenyl phosphate (**12**) shown with linear curve fit which yields k_{cat}/K_m. E) *Sb*-PDE with bis-*p*-nitrophenyl phosphate (**1**). F) *Sb*-PDE with *p*-nitrophenyl phosphate (**11**). G) *Sb*-PDE with diphenyl phosphate (**2**). H) *Sb*-PDE with phenyl phosphate (**12**).

Table S2. Experimental conditions and rate constants with *No*-PDE, *Sb*-PDE and variants.

Enzyme	Substrate	k_{cat} (s^{-1})	K_m (mM)	k_{cat}/K_m ($M^{-1}s^{-1}$)	Enzyme Concentration	Max Substrate Concentration (mM)[a]	Min Substrate Concentration (μM)[a]
No-PDE	Bis-p-nitrophenyl phosphate (**1**)	6.7 ± 0.2	0.62 ± 0.04	$1.08 \pm 0.08 \times 10^4$	47 nM	2.5	47
No-PDE	Bis-phenyl phosphate (**2**)	49 ± 3	2.3 ± 0.2	$2.1 \pm 0.3 \times 10^4$	154 nM	5.5	130
No-PDE	Bis-2-chloroethyl phosphate (**3**)	nd	nd	$7.7 \pm 0.3 \times 10^2$	560 nM	2	na
No-PDE	Bis-1,3-dichloroisopropyl phosphate (**4**)	nd	nd	$1.3 \pm 0.1 \times 10^4$	3.1 nM	2	na
No-PDE	Bis-2,3-dibromopropyl phosphate (**5**)	nd	nd	$4.1 \pm 0.3 \times 10^3$	30 nM	1.2	na
No-PDE	Bis2-butoxyethyl phosphate (**6**)	nd	nd	$1.40 \pm 0.08 \times 10^3$	90 mM	1.1	na
No-PDE	Dibutyl phosphate (**7**)	nd	nd	$1.17 \pm 0.02 \times 10^2$	1 μM	3.8	na
No-PDE	Dicyclohexyl phosphate (**10**)	nd	nd	$6.4 \pm 0.3 \times 10^2$	412 nM	2.0	na
No-PDE	4-Nitrophenyl Phosphate (**11**)	0.70 ± 0.04	1.4 ± 0.2	$5.0 \pm 0.8 \times 10^2$	47 nM	5.0	94
No-PDE	Phenyl Phosphate (**12**)	nd	nd	$3.3 \pm 0.1 \times 10^1$	9.1 μM	5.0	94
No-PDE	2-chloroethyl phosphate (**13**)	NO	NO	$< 1 \times 10^0$	1 μM	2.2	na
No-PDE	1,3-dichloroisopropyl phosphate (**14**)	nd	nd	$< 1 \times 10^0$	1 μM	1.6	na
No-PDE	2,3-dibromopropyl phosphate (**15**)	NO	NO	$< 1 \times 10^0$	1 μM	1.0	na
No-PDE	2-butoxyethl phosphate (**16**)	NO	NO	$< 1 \times 10^0$	1 μM	1.5	na
No-PDE	Butyl phosphate (**17**)	nd	nd	$< 5 \times 10^0$	1 μM	2.4	na
No-PDE	Cyclohexyl phosphate **(18)**	NO	NO	$< 1 \times 10^0$	1 μM	1.9	na
Sb-PDE	Bis-p-nitrophenyl phosphate (**1**)	19.8 ± 0.6	0.14 ± 0.02	$1.4 \pm 0.2 \times 10^5$	15 nM	2.5	48
Sb-PDE	Bis-phenyl phosphate (**2**)	49 ± 4	0.9 ± 0.1	$5.0 \pm 0.3 \times 10^4$	149 nM	1.4	26
Sb-PDE	Bis-2-chloroethyl phosphate (**3**)	nd	nd	$3.9 \pm 0.2 \times 10^1$	10 μM	1.0	na
Sb-PDE	Bis-1,3-dichloroisopropyl phosphate (**4**)	nd	nd	$2.8 \pm 0.2 \times 10^3$	2.25 nM	2	na
Sb-PDE	Bis-2,3-dibromopropyl phosphate (**5**)	nd	nd	$3.4 \pm 0.3 \times 10^3$	25 nM	1.3	na
Sb-PDE	Bis2-butoxyethyl phosphate (**6**)	nd	nd	$7.0 \pm 0.3 \times 10^2$	180 nM	2	na
Sb-PDE	Dibutyl phosphate (**7**)	nd	nd	$3.49 \pm 0.09 \times 10^1$	6 μM	2	na
Sb-PDE	Dicyclohexyl phosphate (**10**)	nd	nd	$8 \pm 1 \times 10^2$	5 μM	2.0	na
Sb-PDE	4-Nitrophenyl Phosphate (**11**)	0.63 ± 0.02	0.14 ± 0.01	$4.4 \pm 0.4 \times 10^3$	149 nM	2.6	48
Sb-PDE	Phenyl Phosphate (**12**)	0.213 ± 0.008	1.36 ± 0.09	$1.6 \pm 0.1 \times 10^2$	1.5 μM	2.0	38
Sb-PDE	2-chloroethyl phosphate (**13**)	NO	NO	$< 1 \times 10^0$	1 μM	2.2	na
Sb-PDE	1,3-dichloroisopropyl phosphate (**14**)	NO	NO	$< 1 \times 10^0$	1 μM	1.6	na
Sb-PDE	2-butoxyethl phosphate (**15**)	NO	NO	$< 1 \times 10^0$	1 μM	1.0	na
Sb-PDE	2-butoxyethl phosphate (**16**)	NO	NO	$< 1 \times 10^0$	1 μM	1.5	na
Sb-PDE	Butyl phosphate (**17**)	NO	NO	$< 1 \times 10^0$	1 μM	2.4	na
Sb-PDE	Cyclohexyl phosphate **(18)**	NO	NO	$< 1 \times 10^0$	1 μM	1.9	na

nd = not determined. NO = no reaction observed. [a]Titration curves used serial dilutions to obtain 32 points evenly spaced from highest to lowest concentrations given. Reactions followed by ^{31}P NMR used only a single concentration given as the high concentration.

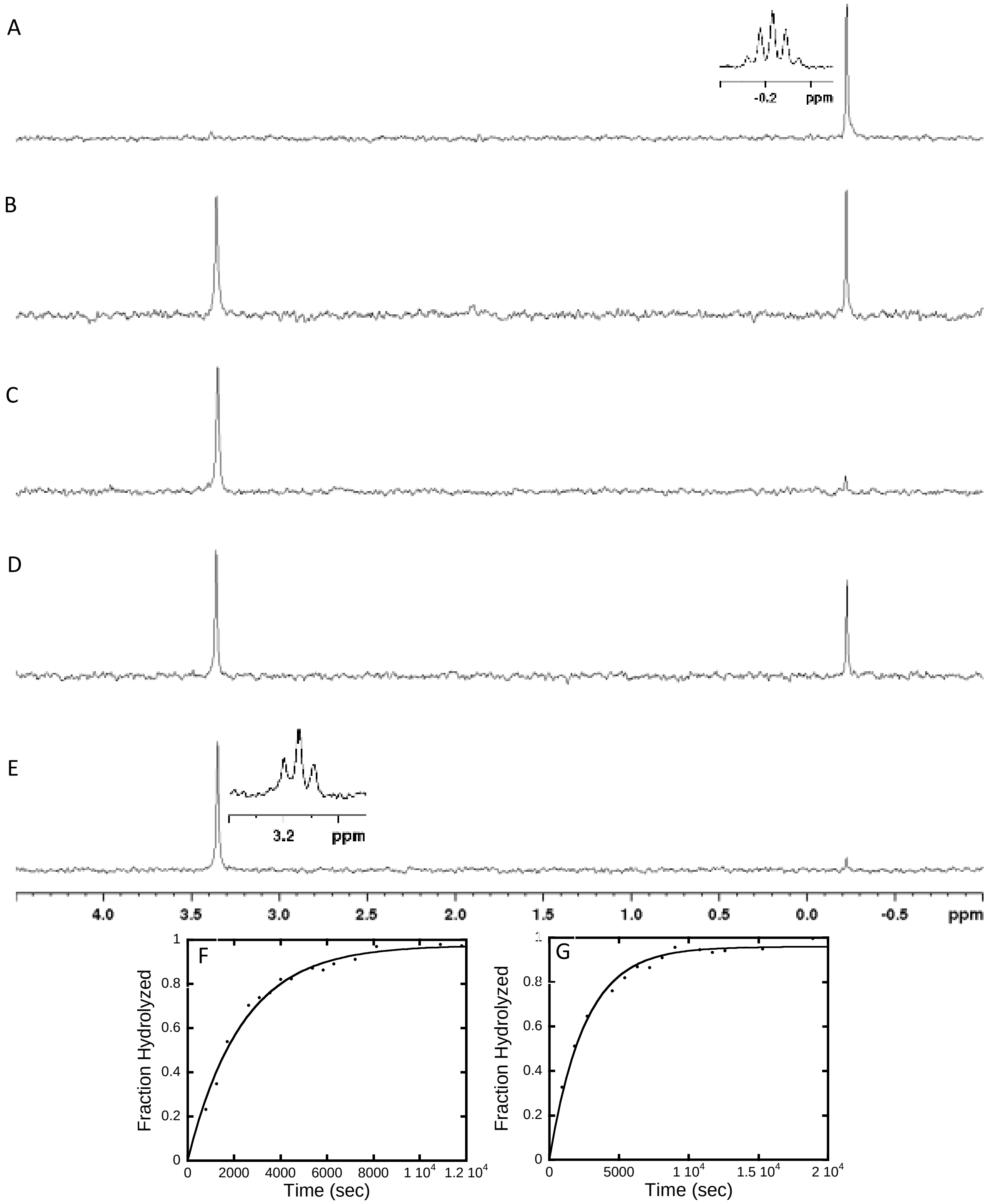

Figure S2: Hydrolysis of bis-2-chloroethyl phosphate (**3**) by *No*-PDE and *Sb*-PDE. A) ^{31}P NMR spectra of 2 mM bis-2-chloroethyl phosphate (**3**) in 50 mM Hepes pH 8.0. Inset is the ^{1}H coupled spectra showing the expected quintuplet signal. B) Same as (A) after 65 min incubation with 560 nM *No*-PDE. C) Same as (A) after 230 min incubation with 560 nM *No*-PDE. D) Same as (A) after 33 min incubation with 10 µM *Sb*-PDE. E) Same as (A) after 138 min incubation with 10 µM *Sb*-PDE. Inset is the ^{1}H coupled spectra showing the expected triplet pattern for 2-chloroethyl phosphate (**3**). Peak at -0.22 ppm is identified as bis-2-chloroethyl phosphate (**3**). Peak at 3.25 ppm is identified as 2-chloroethyl phosphate (**13**). F) Data for hydrolysis by *No*-PDE fit to equation 1. G) Data for hydrolysis by *Sb*-PDE fit to equation 1.

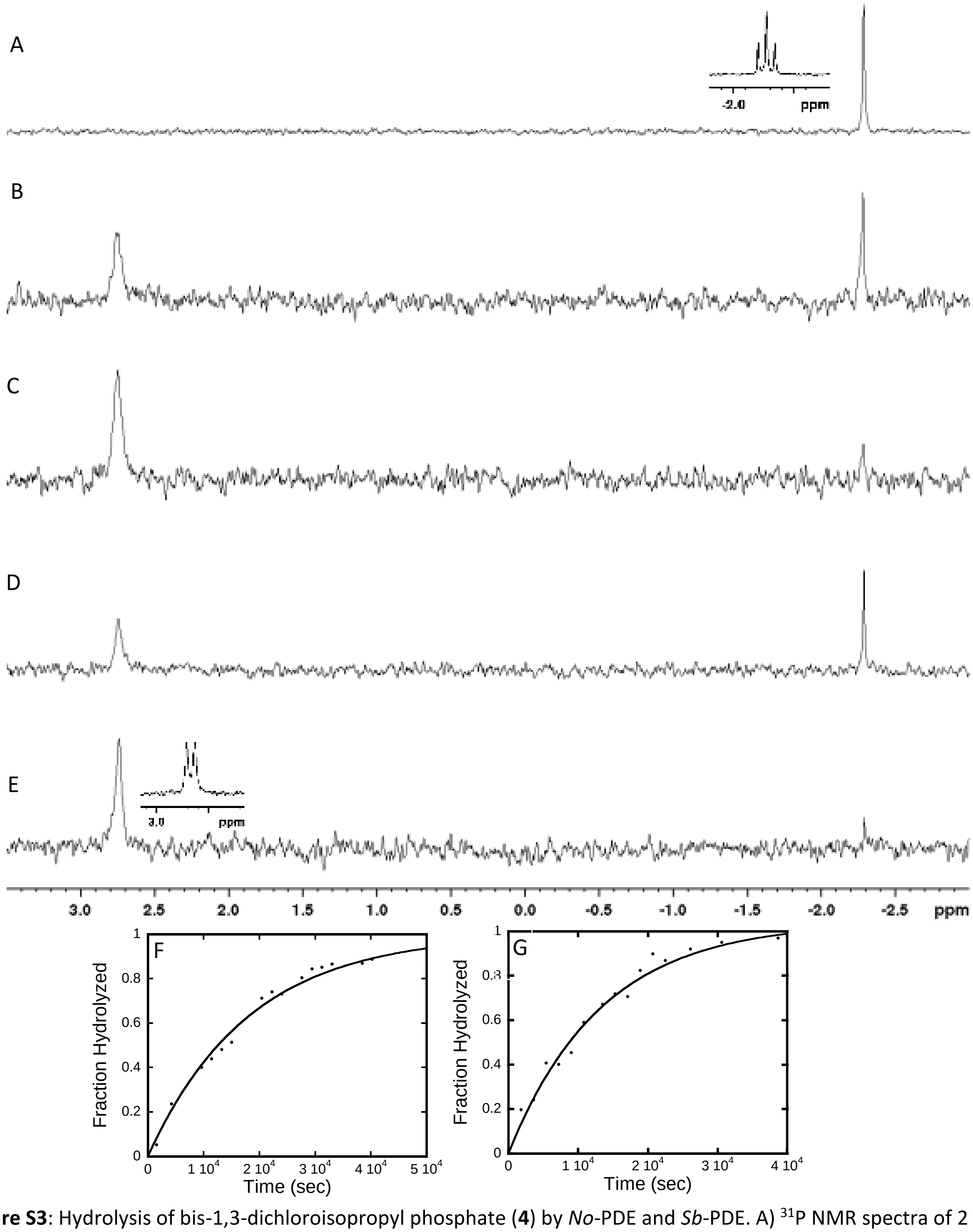

Figure S3: Hydrolysis of bis-1,3-dichloroisopropyl phosphate (**4**) by *No*-PDE and *Sb*-PDE. A) ^{31}P NMR spectra of 2 mM bis-1,3-dichloroisopropyl phosphate (**4**) in 50 mM Hepes pH 8.0. Inset is the ^{1}H coupled spectra showing the expected triplet signal. B) Same as (A) after 330 min incubation with 3.1 nM *No*-PDE. C) Same as (A) after 540 min incubation with 3.1 nM *No*-PDE. D) Same as (A) after 213min incubation with 23 nM *Sb*-PDE. E) Same as (A) after 453 min incubation with 23 nM *Sb*-PDE. Inset is the ^{1}H coupled spectra showing the expected doublet pattern for 1,3-dichloroisopropyl phosphate (**14**). Peak at -2.29 ppm is identified as bis-1,3-dichloroisopropyl phosphate (**4**). Peak at 2.75 ppm is identified as 1,3-dichloroisopropyl phosphate (**14**). F) Data for hydrolysis by *No*-PDE fit to equation 1. G) Data for hydrolysis by *Sb*-PDE fit to equation 1.

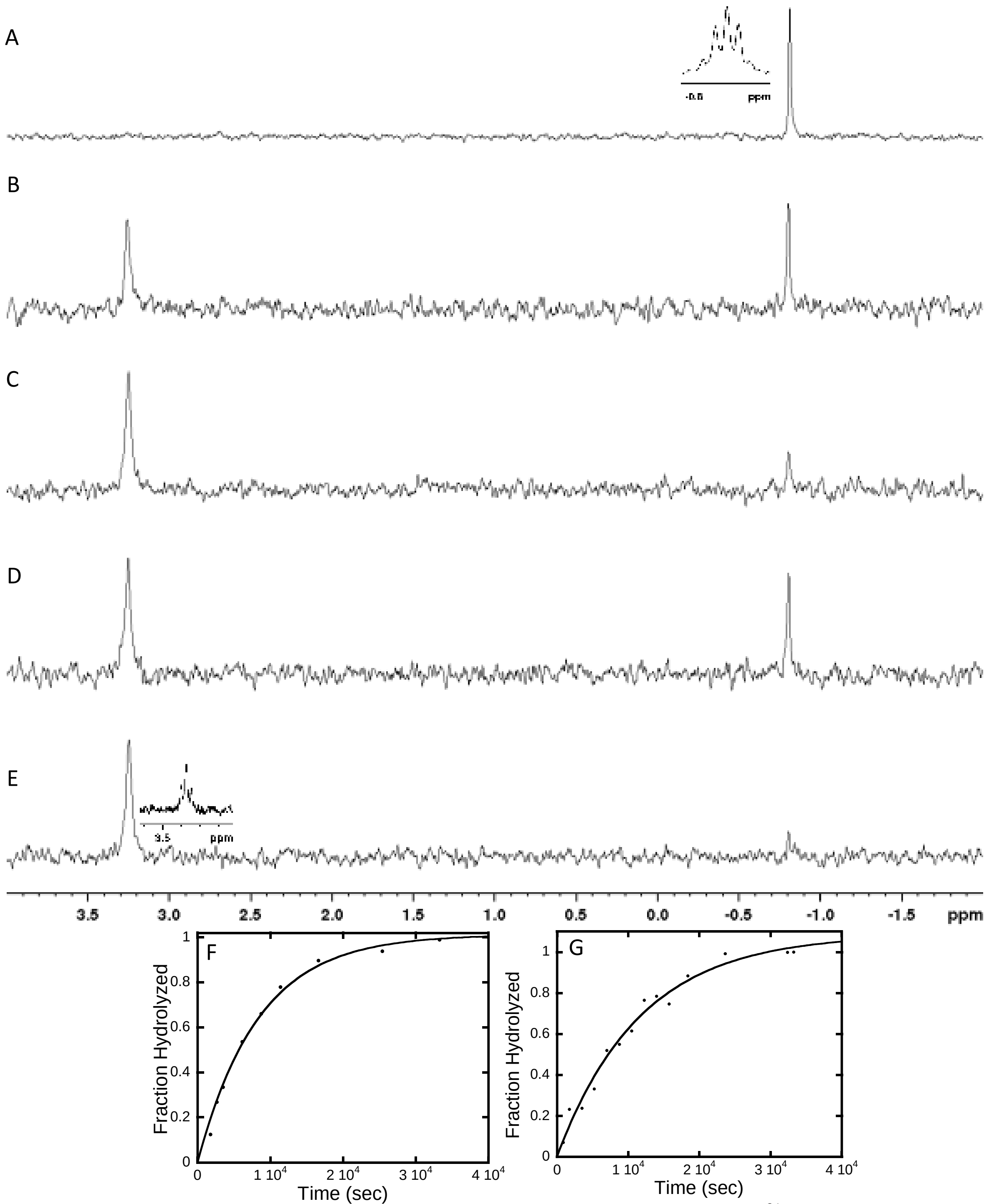

Figure S4: Hydrolysis of bis-2,3-dibromopropyl phosphate (**5**) by *No*-PDE and *Sb*-PDE. A) ^{31}P NMR spectra of 2 mM bis-2,3-dibromopropyl phosphate (**5**) in 50 mM Hepes pH 8.0. Inset is the ^{1}H coupled spectra showing the expected quintuplet signal. B) Same as (A) after 205 min incubation with 30 nM *No*-PDE. C) Same as (A) after 322 min incubation with 30 nM *No*-PDE. D) Same as (A) after 270min incubation with 25 nM *Sb*-PDE. E) Same as (A) after 395 min incubation with 25 nM *Sb*-PDE. Inset is the ^{1}H coupled spectra showing the expected triplet pattern for 2,3-dibromopropyl phosphate (**15**). Peak at -0.81 ppm is identified as bis-2,3-dibromopropyl phosphate (**5**). Peak at 3.25 ppm is identified as 2,3-dibromopropyl phosphate (**15**). F) Data for hydrolysis by *No*-PDE fit to equation 1. G) Data for hydrolysis by *Sb*-PDE fit to equation 1.

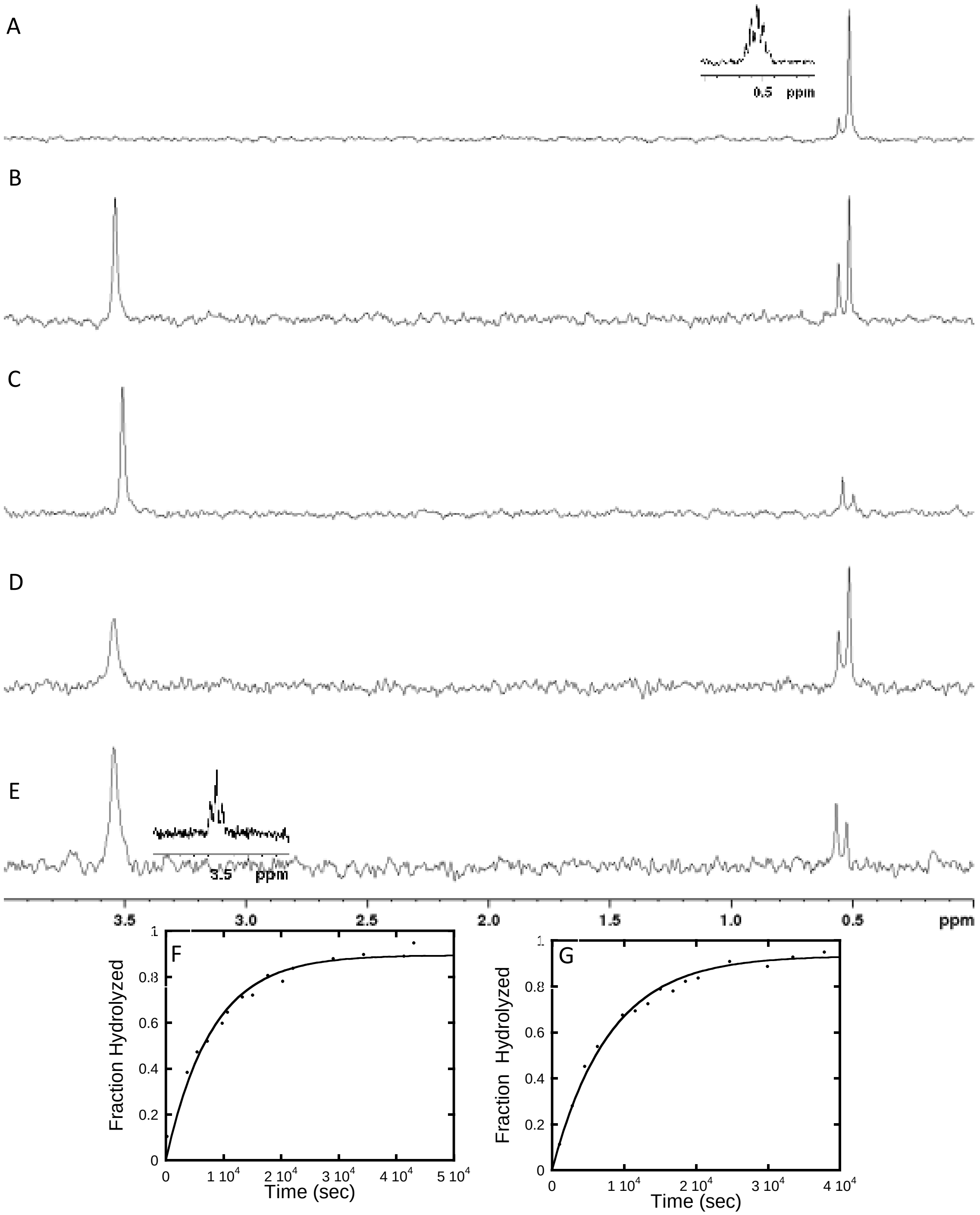

Figure S5: Hydrolysis of bis-2-butoxyethyl phosphate (**6**) by *No*-PDE and *Sb*-PDE. A) ^{31}P NMR spectra of 2 mM bis-2-butoxyethyl phosphate (**6**) in 50 mM Hepes pH 8.0. Inset is the ^{1}H coupled spectra showing the expected quintuplet signal. B) Same as (A) after 205 min incubation with 90 nM *No*-PDE. C) Same as (A) after 322 min incubation with 90 nM *No*-PDE. D) Same as (A) after 270min incubation with 180 nM *Sb*-PDE. E) Same as (A) after 395 min incubation with 180 nM *Sb*-PDE. Inset is the ^{1}H coupled spectra showing the expected triplet pattern for 2-butoxyethyl phosphate (**16**). Peak at 0.52 ppm is identified as bis-2-butoxyethyl phosphate (**6**). Peak at 3.55 ppm is identified as 2-butoxyethyl phosphate (**16**). The peak at 0.56 ppm is an unidentified non-hydrolyzed contaminant. F) Data for hydrolysis by *No*-PDE fit to equation 1. G) Data for hydrolysis by *Sb*-PDE fit to equation 1.

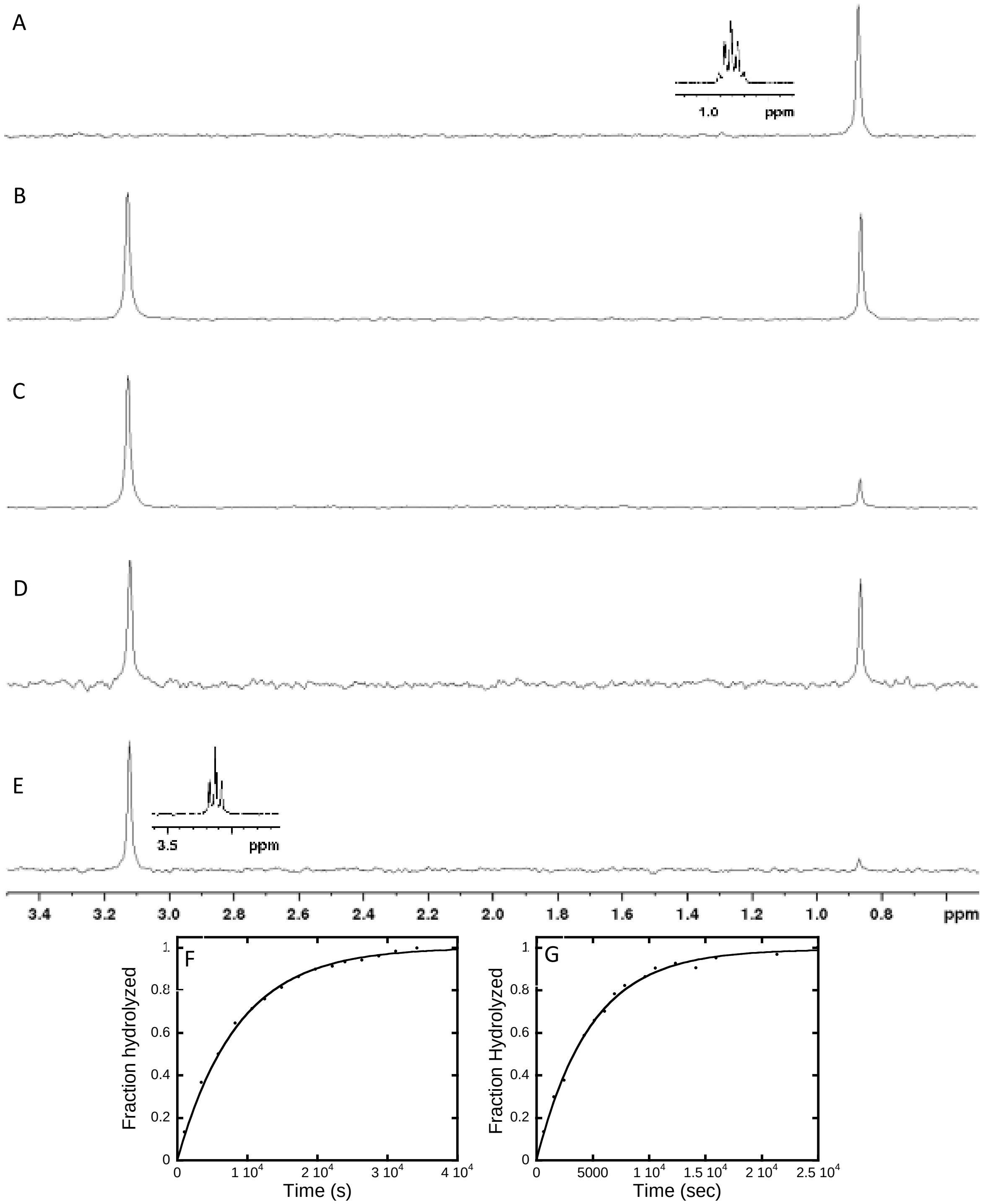

Figure S6: Hydrolysis of dibutyl phosphate (**7**) by *No*-PDE and *Sb*-PDE. A) ^{31}P NMR spectra of 2 mM dibutyl phosphate (**7**) in 50 mM Hepes pH 8.0. Inset is the ^{1}H coupled spectra showing the expected quintuplet signal. B) Same as (A) after 315 min incubation with 1 µM *No*-PDE. C) Same as (A) after 604 min incubation with 1 µM *No*-PDE. D) Same as (A) after 79min incubation with 6 µM *Sb*-PDE. E) Same as (A) after 259 min incubation with 6 µM *Sb*-PDE. Inset is the ^{1}H coupled spectra showing the expected triplet pattern for butyl phosphate (**17**). Peak at 0.86 ppm is identified as dibutyl phosphate (**7**). Peak at 3.12 ppm is identified as butyl phosphate (**17**). F) Data for hydrolysis by *No*-PDE fit to equation 1. G) Data for hydrolysis by *Sb*-PDE fit to equation 1.

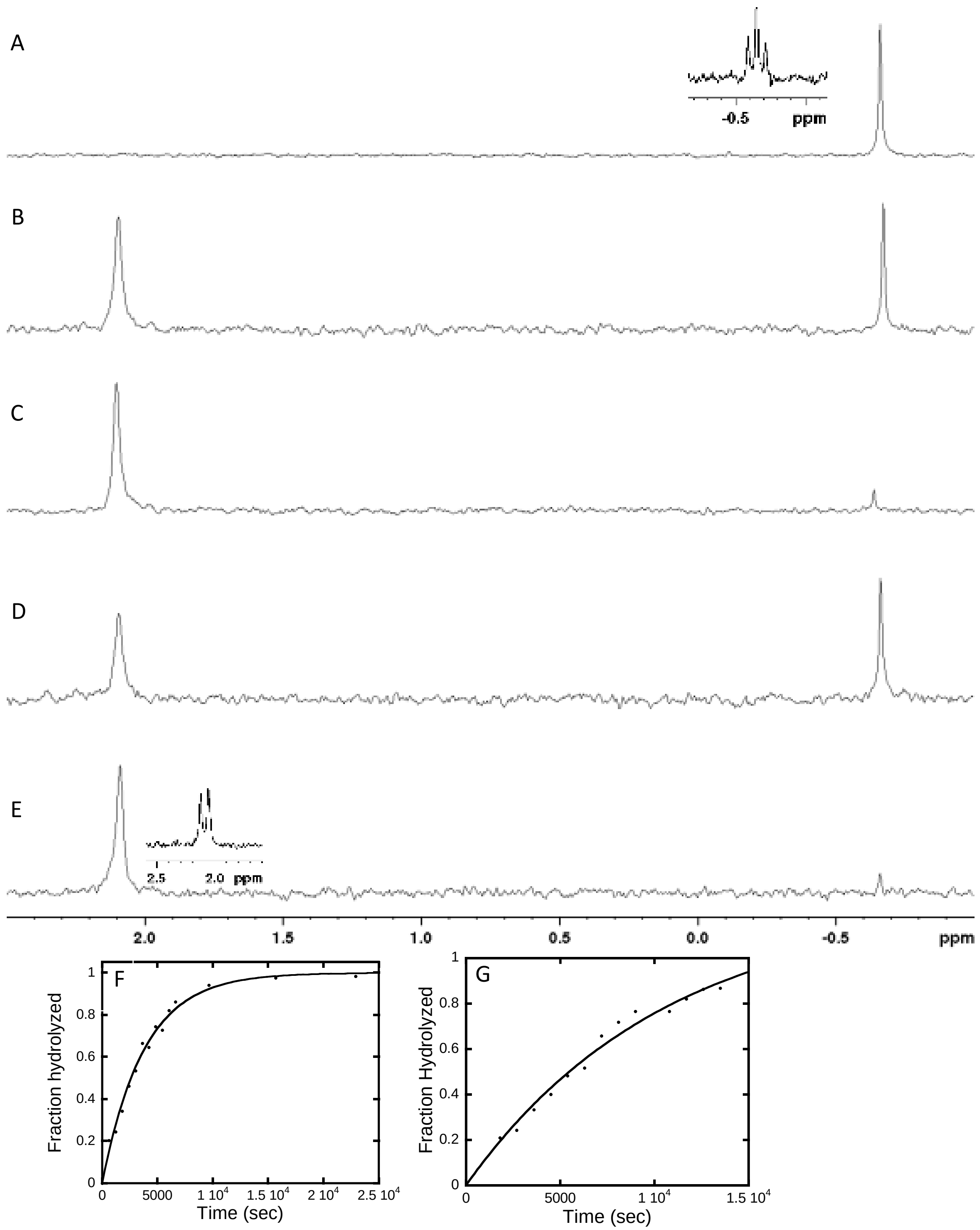

Figure S7: Hydrolysis of dicyclohexyl phosphate (**10**) by *No*-PDE and *Sb*-PDE. A) ^{31}P NMR spectra of 2 mM dicyclohexyl phosphate (**10**) in 50 mM Hepes pH 8.0. Inset is the ^{1}H coupled spectra showing the expected triplet signal. B) Same as (A) after 240 min incubation with 410 nM *No*-PDE. C) Same as (A) after 390 min incubation with 410 nM *No*-PDE. D) Same as (A) after 65 min incubation with 5 µM *Sb*-PDE. E) Same as (A) after 110 min incubation with 5 µM *Sb*-PDE. Inset is the ^{1}H coupled spectra showing the expected doublet pattern for cyclohexyl phosphate (**18**). Peak at -0.66 ppm is identified as dicyclohexyl phosphate (**10**). Peak at 2.09 ppm is identified as cyclohexyl phosphate (**18**). F) Data for hydrolysis by *No*-PDE fit to equation 1. G) Data for hydrolysis by *Sb*-PDE fit to equation 1.

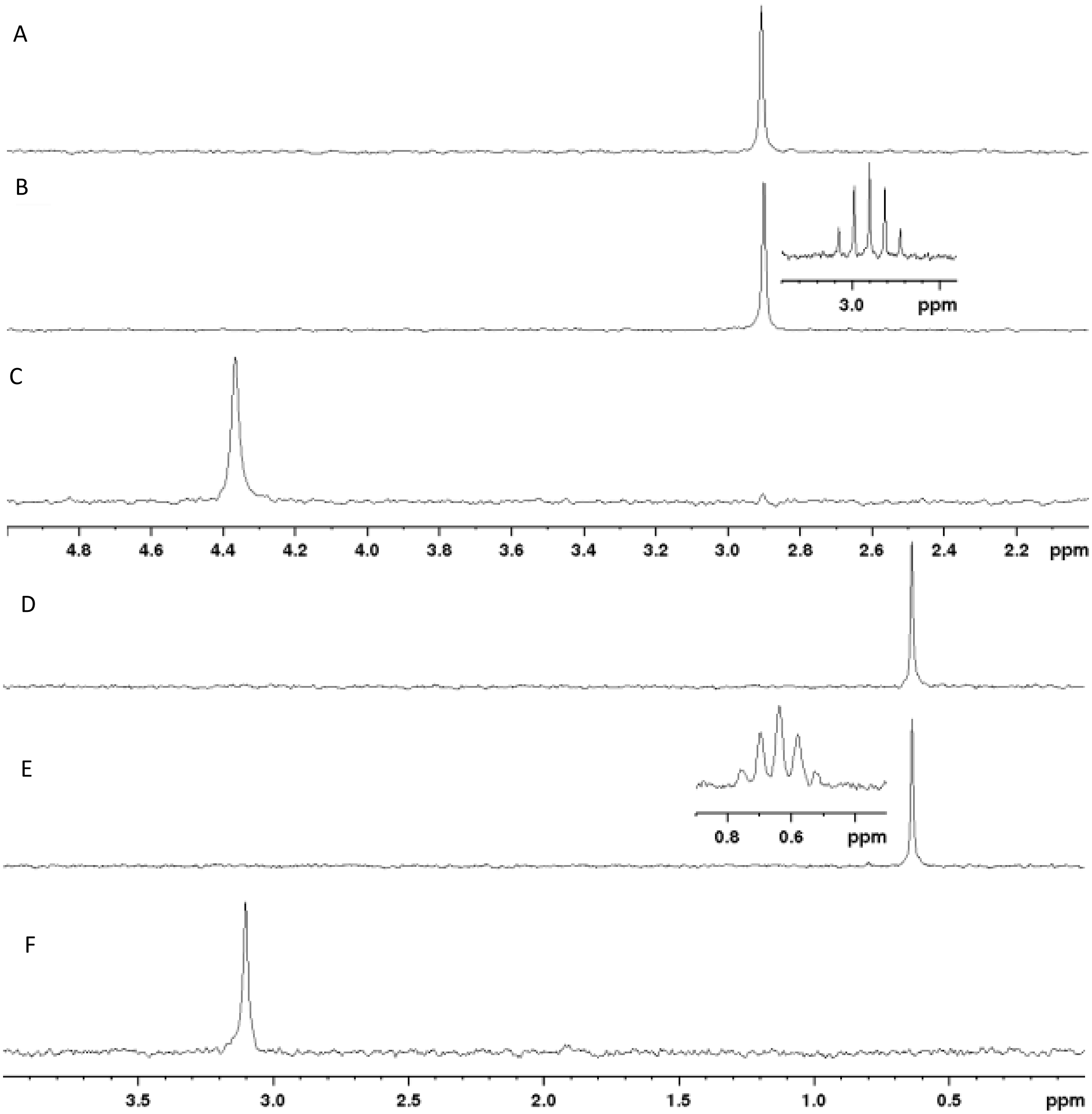

Figure S8: A) ^{31}P NMR spectra of 4 mM dimethyl phosphate (**9**). B) ^{31}P NMR spectra of 4 mM dimethyl phosphate after 72 hr incubation with 1 µM *No*-PDE. Inset shows proton coupled spectrum of sample with expected splitting from dimethyl phosphate (**9**). C) ^{31}P NMR spectra of 2.5 mM chemically prepared methyl phosphate. D) ^{31}P NMR spectra of 3.4 mM diethyl phosphate (**8**). E) ^{31}P NMR spectra of 3.4 mM dimethyl phosphate (**8**) after 72 hr incubation with 1 µM No-PDE. Inset shows proton coupled spectrum of sample with expected splitting from diethyl phosphate (**8**). F) ^{31}P NMR spectra of 2.5 mM chemically prepared ethyl phosphate. Similar results were obtained with *Sb*-PDE (data not shown)

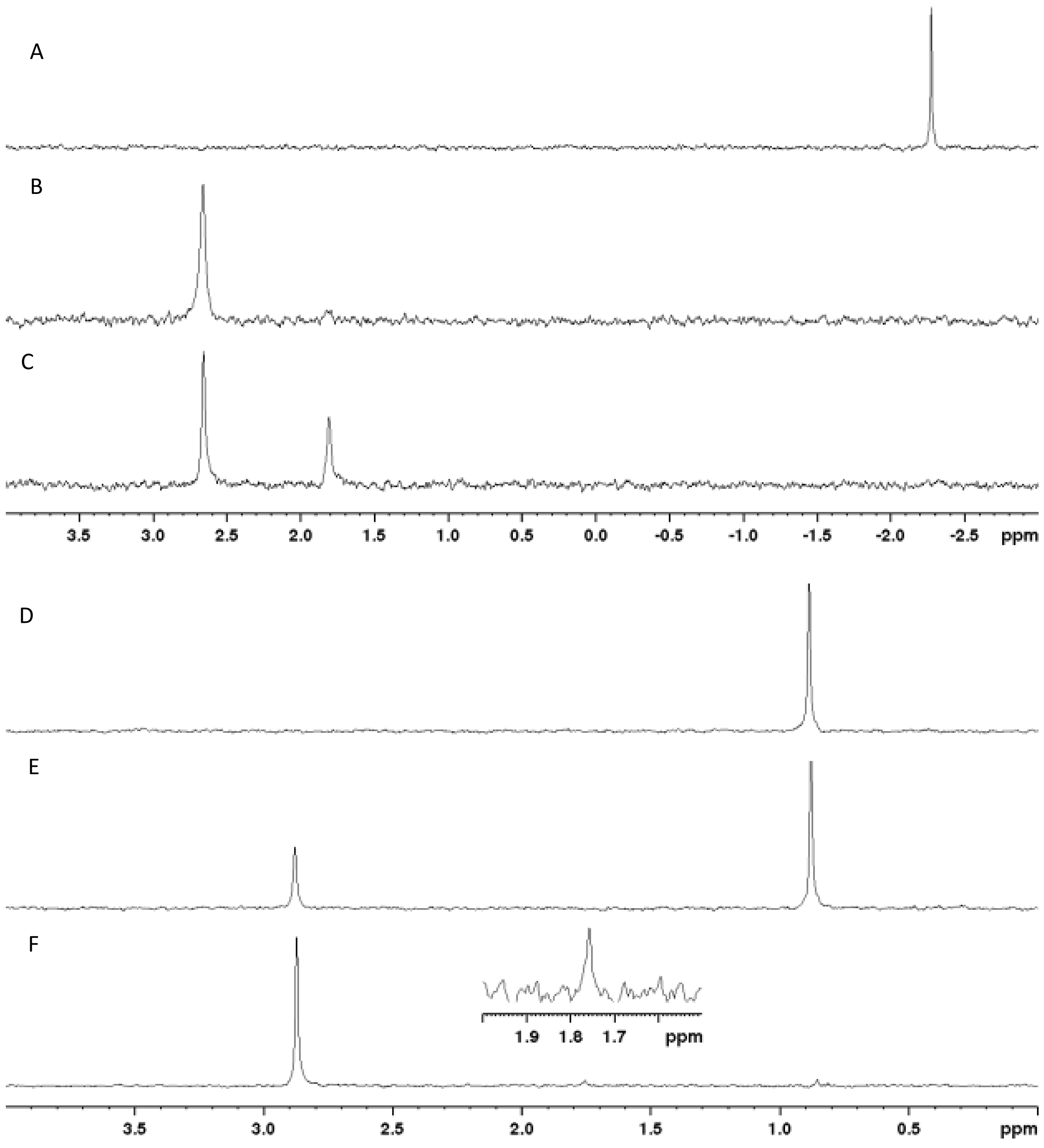

Figure S9: Hydrolysis of bis-1,3-dichloroisopropyl phosphate (**4**) by *No*-PDE. A) ^{31}P NMR spectra of 1.6 mM bis-1,3-dichloroisopropyl phosphate (**4**) in 50 mM Hepes pH 8.0. B) Same as (A) after 3 hr incubation with 1 µM *No*-PDE. C) Same as (A) after 72 hr incubation with 1 µM *No*-PDE. Peak at -2.28 ppm is identified as bis-1,3-dichloroisopropyl phosphate (**4**). Peak at 2.66 ppm is identified as 1,3-dichloroisopropyl phosphate (**14**). The peak at 1.76 ppm in spectrum C is identified as phosphate. D) ^{31}P NMR spectra of 2.4 mM dibutyl phosphate (**7**) in 50 mM Hepes pH 8.0. E) Same as D after 1 hr incubation with 1 µM *No*-PDE. F) Same as (A) after 48 hr incubation with 1 µM *No*-PDE. Peak at 0.85 ppm is identified as dibutyl phosphate (**7**). Peak at 2.87 ppm is identified as butyl phosphate (**17**). The inset in F shows the peak at 1.76 ppm identified as phosphate. Similar experiments with *Sb*-PDE failed to show any phosphate product (data not shown)

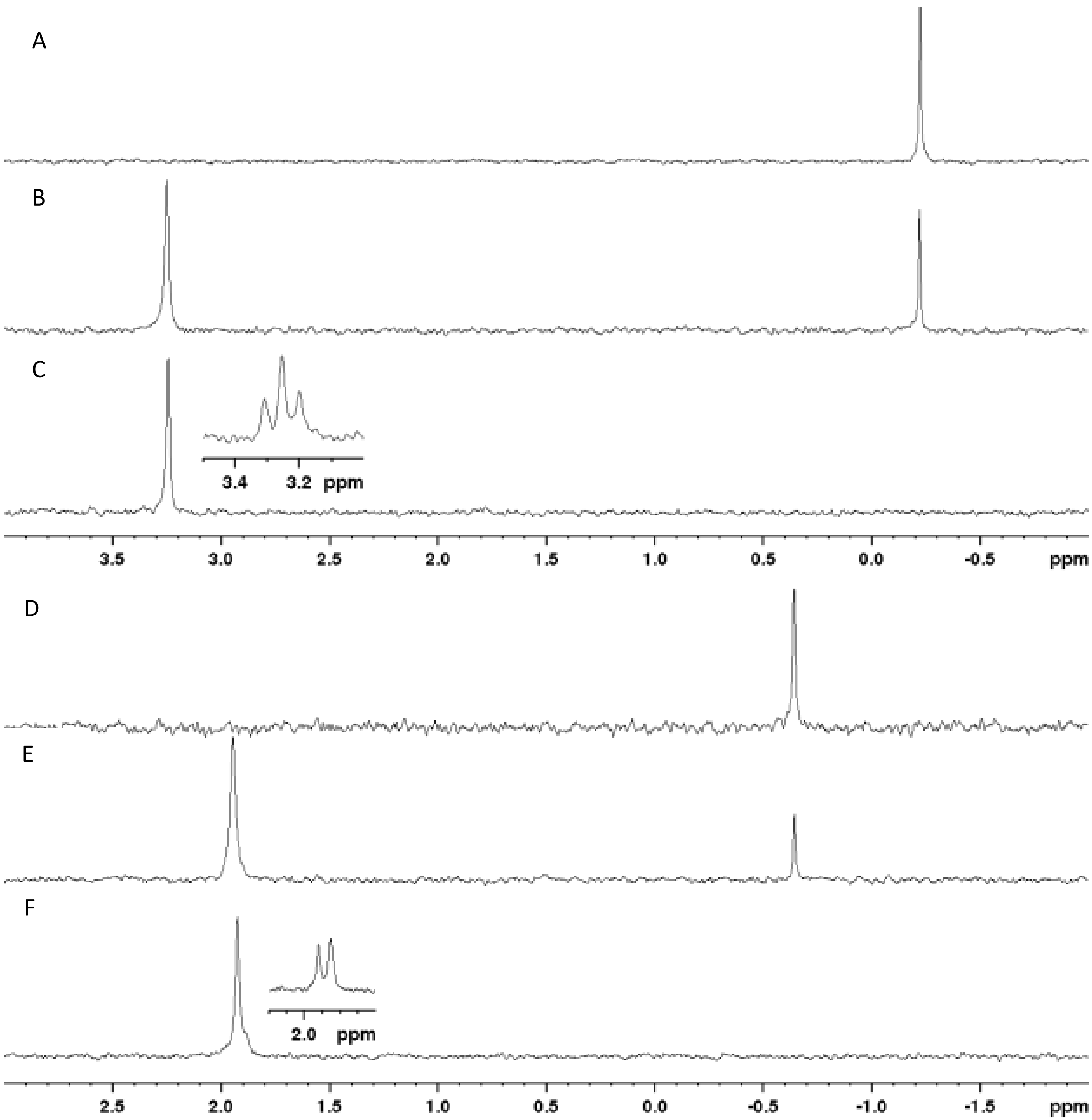

Figure S10: Hydrolysis of bis-2-chloroethyl phosphate (**3**) by *No*-PDE. A) ^{31}P NMR spectra of 2.2 mM bis-2-dichloroethyl phosphate (**3**) in 50 mM Hepes pH 8.0. B) Same as (A) after 2 hr incubation with 1 µM *No*-PDE. C) Same as (A) after 72 hr incubation with 1 µM *No*-PDE. Inset shows proton coupled spectra with expected splitting for 2-chloroethyl phosphate (**13**). Peak at -0.22 ppm is identified as bis-2-dichloroethyl phosphate (**3**). Peak at 3.29 ppm is identified as 2-chloroethyl phosphate (**13**). D) ^{31}P NMR spectra of 1.9 mM dicyclohexyl phosphate (**10**) in 50 mM Hepes pH 8.0. E) Same as D after 2 hr incubation with 1 µM *No*-PDE. F) Same as (D) after 72 hr incubation with 1 µM *No*-PDE. Peak at - 0.64 ppm is identified as dicyclohexyl phosphate (**10**). Peak at 1.93 ppm is identified as cyclohexyl phosphate (**18**). The inset in F shows proton coupled spectra with splitting expected for cyclohexyl phosphate (**18**) peaks are at 1.92 ppm and 1.85 ppm. Absent from spectra is a singlet at 1.76 ppm due to phosphate. Similar experiments compounds **15**-**17** and compounds **13**-**18** with *Sb*-PDE failed to show any phosphate product (data not shown).

A

```
pdb:3E38 -----------QRRNEIQVPDLDGYTTLKCDFHMHSVFSDGLVWPTVRVDEAYRDGLDAISLTEHIEYRP  81
No-PDE   EFVTQSDQRPPSPARLIFPDTKDGHHIFAGDLHLHTVFSDGSAWPTLRLAEAAHDGLKFVSFTEHDIITP  98
Sb-PDE   EFVTQSDQRPPSPARLIFPDTKDGHHIFAGDLHLHTVFSDGSAWPTLRLAEAAHDGLKFVSFTEHDIITP  98
                    .... *  .  . **:.....*.*:*.***** .***.*..** .***. :*.***   .*

pdb:3E38 HKQDVVSDHNRSFDLCREQAE-----KLGILLIKGSEITRAMAPGHFNAIFLSDSNPL------------  134
No-PDE   KIRDTGTNKNRSYEIGRAFISSGMLGLGGMKLSLGSEITRA--IGHFNCHFLKDANALNPGKVHYSTFKQ  166
Sb-PDE   KIRDTGTNKNRSYEIGRAFISSGMLGLGGMKLSLGSEITRA--IGHFNCHFLKDANALNPGKVHYSTFKQ  166
         .  .*.  ...***::..*.  ..       *:  *.  *******    ****.  **.*.*.*

pdb:3E38 --------------EQKDYKDAFREAKKQGAFMFWNHPGWDSQQPDTTKWWPEHTALYQEGCMHGIEVAN  190
No-PDE   TVALDRVDWNRTSADEKDLEARFREAKRQGAICQWNHPTIPSSSPGEAVVTPFLERMFSEGLLSGIEVAQ  236
Sb-PDE   TGGQLRVDWNRTSADEKDLEARFREAKRQGAICQWNHPTIPSSSPGEAVVTPFLERMFSEGLLSGIEVAQ  236
                       :.**....*****:***.   ****.   *..*...     *   ..::.** :.*****.

pdb:3E38 GHLYMPEAIQWCLDKNLTMIGTSDIHQPIQTDYDFEKGEHRTMTFVFAKERSLQGIREALDNRRTAAYFH  260
No-PDE   SDFIHPSAMDVALKYNLYIAATTDAHLGTALEQEAAGMDHRVATLFLTQGEDEAAFKDALEKRRTVGLFR  306
Sb-PDE   SDFIHPSAMDVALKYNLYIAATTDAHLGTALEQEAAGMDHRVATLFLTQGEDEAAFKDALEKRRTVGLFR  306
         ...   *.*:.  .*.  **  :..*.*.*  ...  .:.:  ..  :**..*...........::**:.***...*.

pdb:3E38 ELLIGREDLLRPFFEKCVKIEEVSRNEQGV------TLSITNVTDLVLKLKKTAHDTLLVYFRDMTLKPH  324
No-PDE   DTFFGTRENVQTVMNAVLKMSFTKYSPSEFLIGNSGEMEIANAGPIDVELEIVQPDFRLLNYPRFVKIPH  376
Sb-PDE   DTFFGTRENVQTVMNAVLKMSFTKYSPSEFLIGNSGEMEIANAGPIDVELEIVQPDFRLLNYPRFVKIPH  376
         :...*..: .......  .*:.  ..  .  ...       .:.*.*..  .  ..*.  ...*   *..:....   **

pdb:3E38 TR-YTVRIGFKQGIKGGDVNFEVTNFIVAPDKGLKYTISL-------------------------  363
No-PDE   GSKVTLKALRIDGPNFKGLKVRVINSLVTSRDQLEFFLEAQPFDLNAITGKGTLSNIPDDLDGWM  441
Sb-PDE   GSKVTLKALRIDGPNFKGLKVRVINSLVTSRDQLEFFLEAQPFDLNAITGKGTLSNIPDDLDGWM  441
         ..   *.:.    .*  .  ......*.*  .*.....*.:  ...
```

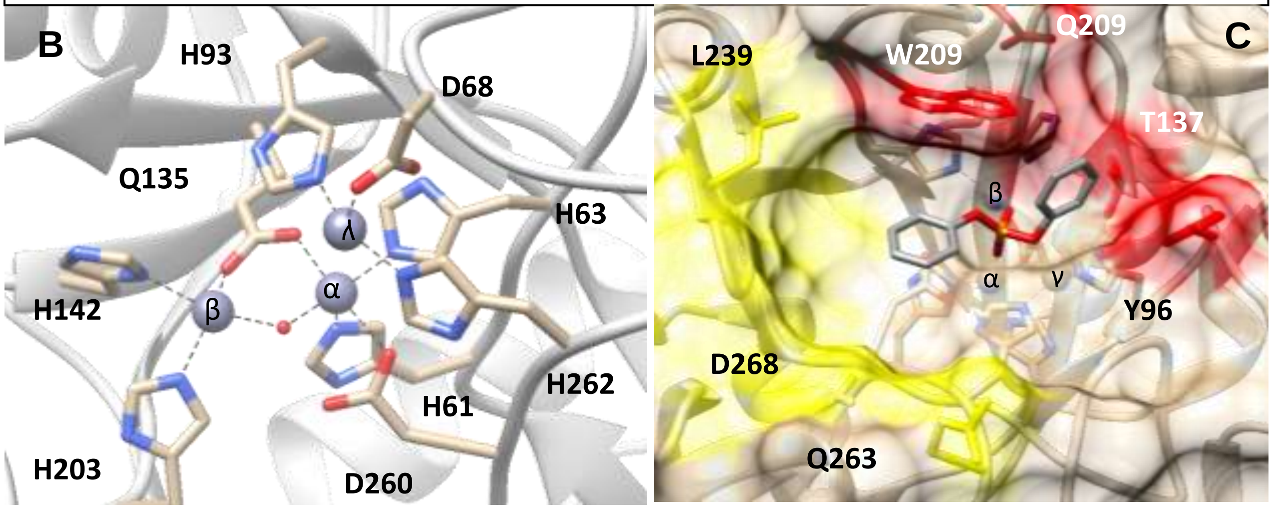

Figure S11: A) Clustal W sequence alignment between *Sb*-PDE, *No*-PDE and the only homolog of known structure, pdb:3E38. Metal ligands for the trinuclear metal center are highlighted in green. Side ester binding pocket residues are highlighted in yellow and leaving group pocket residues are highlighted in red. B) Trinuclear metal center in pdb:3E38 (numbering from No-PDE). C) Surface of active site in pdb:3E38 with diphenyl phosphate docked. The phosphate oxygens bind in a bidentate manner to the α- and β-metals. The leaving group is identified as the ester group within binding distance to the γ-metal which acts as a Lewis acid to the leaving group in the mechanism of PHP enzymes. The bottom of the binding pocket is lined by the metal ligands. The leaving group extends toward the pocket between residues Y96, T137, W206, and Q209 (shown in red). The side ester of the substrate extends into the pocket line by Q236, L239, and D268 (colored yellow).

Table S3: Kinetic constants for variants of *No*-PDE with aromatic di- and monoesters. Exact conditions and errors are presented in Table S4.

	Compound 1			Compound 11			Compound 2			Compound 12		
Variant	k_{cat} (s^{-1})	K_m (mM)	k_{cat}/K_m ($M^{-1}s^{-1}$)	k_{cat} (s^{-1})	K_m (mM)	k_{cat}/K_m ($M^{-1}s^{-1}$)	k_{cat} (s^{-1})	K_m (mM)	k_{cat}/K_m ($M^{-1}s^{-1}$)	k_{cat} (s^{-1})	K_m (mM)	k_{cat}/K_m ($M^{-1}s^{-1}$)
WT	6.7 ± 0.2	0.62 ± 0.04	$1.08 \pm 0.08 \times 10^4$	0.70 ± 0.04	1.4 ± 0.2	$5.0 \pm 0.8 \times 10^2$	49 ± 3	2.3 ± 0.2	$2.1 \pm 0.3 \times 10^4$	nd	nd	$3.3 \pm 0.1 \times 10^1$
I96Y	nd	nd	$3.5 \pm 0.1 \times 10^2$	0.21 ± 0.01	2.11 ± 0.08	$1.0 \pm 0.1 \times 10^2$	nd	nd	$6.6 \pm 0.4 \times 10^2$	nd	nd	$< 10^0$
I96A	19 ± 2	0.49 ± 0.06	$3.9 \pm 0.6 \times 10^4$	1.0 ± 0.1	5.0 ± 0.5	$1.82 \pm 0.03 \times 10^2$	56 ± 8	5 ± 1	$9.3 \pm 0.2 \times 10^3$	0.20 ± 0.04	13 ± 3	$1.7 \pm 0.6 \times 10^1$
T137A	1.89 ± 0.06	0.77 ± 0.06	$2.3 \pm 0.2 \times 10^3$	0.09 ± 0.01	3.7 ± 0.4	$2.4 \times 10^1 \pm 0.3$	12 ± 2	3.3 ± 0.7	$3.6 \pm 0.9 \times 10^3$	nd	nd	$< 10^0$
I206W	0.60 ± 0.01	0.05 ± 0.004	$1.4 \pm 0.1 \times 10^4$	0.30 ± 0.01	0.22 ± 0.02	$1.4 \pm 0.1 \times 10^3$	5.0 ± 0.2	0.88 ± 0.09	$5.6 \pm 0.7 \times 10^3$	0.21 ± 0.01	0.37 ± 0.04	$5.8 \pm 0.7 \times 10^2$
I206A	nd	nd	$2.82 \pm 0.05 \times 10^3$	1.83 ± 0.06	1.4 ± 0.1	$1.3 \pm 0.1 \times 10^3$	85 ± 7	6.2 ± 0.7	$1.4 \pm 0.2 \times 10^4$	nd	nd	$8.9 \pm 0.2 \times 10^1$
S209Q	6.2 ± 0.3	0.30 ± 0.03	$2.2 \pm 0.3 \times 10^4$	0.60 ± 0.02	0.60 ± 0.07	$1.0 \pm 0.1 \times 10^3$	77 ± 3	2.1 ± .2	$3.7 \pm 0.3 \times 10^4$	nd	nd	$7.9 \pm 0.2 \times 10^1$
S209A	10.9 ± 0.5	0.90 ± 0.08	$1.2 \pm 0.1 \times 10^4$	1.09 ± 0.09	2.32 ± 0.09	$4.7 \pm 0.2 \times 10^2$	65 ± 7	1.7 ± 0.4	$3.5 \pm 0.1 \times 10^4$	nd	nd	$3.7 \pm 0.1 \times 10^1$
F239L	nd	nd	$2.30 \pm 0.03 \times 10^3$	1.34 ± 0.03	2.7 ± 0.1	$4.9 \pm 0.2 \times 10^2$	68 ± 8	4.0 ± 0.8	$1.7 \pm 0.4 \times 10^4$	nd	nd	$2.9 \pm 0.1 \times 10^1$
F239A	6.8 ± 0.4	4.3 ± 0.4	$1.6 \pm 0.2 \times 10^3$	1.50 ± 0.04	2.2 ± 0.1	$6.7 \pm 0.3 \times 10^2$	nd	nd	$4.13 \pm 0.01 \times 10^3$	1.8 ± 0.1	17 ± 1	$1.0 \pm 0.1 \times 10^2$
L263Q	8.7 ± 035	4.9 ± 0.4	$1.8 \pm 0.2 \times 10^3$	1.7 ± 0.1	13 ± 1	$1.3 \pm 0.2 \times 10^2$	nd	nd	$1.05 \pm 0.01 \times 10^4$	nd	nd	$1.15 \pm 0.02 \times 10^1$
L263A	nd	nd	$1.08 \pm 0.01 \times 10^3$	5.8 ± 0.3	6.3 ± 0.4	$9.3 \pm 0.8 \times 10^2$	nd	nd	$9.8 \pm 0.1 \times 10^3$	nd	nd	$3.5 \pm 0.1 \times 10^1$
E268D	nd	nd	$9.6 \pm 0.1 \times 10^2$	0.83 ± 0.03	4.8 ± 0.2	$1.7 \pm 0.1 \times 10^2$	nd	nd	$2.0 \pm 0.05 \times 10^3$	nd	nd	$1.1 \pm 0.02 \times 10^1$
E268A	16.9 ± 0.8	2.5 ± 0.2	$6.7 \pm 0.06 \times 10^3$	1.87 ± 0.07	1.8 ± 0.01	$1.0 \pm 0.1 \times 10^3$	70 ± 6	8.1 ± .9	$8.0 \pm 0.2 \times 10^3$	nd	nd	$6.4 \pm 0.2 \times 10^1$

nd = not determined.

Table S4. Experimental conditions and rate constants with No-PDE, Sb-PDE and variants.

Enzyme	Substrate	Enzyme Concentration	Max Substrate Concentration (mM)[a]	Min Substrate Concentration (µM)
No-PDE I96Y	Bis-p-nitrophenyl Phosphate (**1**)	42 nM	2.5	47
No-PDE I96Y	Bis-phenyl Phosphate (**2**)	418 nM	2.5	47
No-PDE I96Y	4-Nitrophenyl Phosphate (**11**)	84 nM	5.32	67
No-PDE I96Y	Phenyl Phosphate (**12**)	4.2 µM	5	
No-PDE I96A	Bis-p-nitrophenyl Phosphate (**1**)	23 nM	0.2	1
No-PDE I96A	Bis-phenyl Phosphate (**2**)	96 nM	3.4	64
No-PDE I96A	4-Nitrophenyl Phosphate (**11**)	231 nM	5.32	17
No-PDE I96A	Phenyl Phosphate (**12**)	1.9 µM	5	94
No-PDE T137A	Bis-p-nitrophenyl Phosphate (**1**)	27 nM	2.4	8
No-PDE T137A	Bis-phenyl Phosphate (**2**)	225 nM	3.4	64
No-PDE T137A	4-Nitrophenyl Phosphate (**11**)	2.7 µM	5.2	16
No-PDE T137A	Phenyl Phosphate (**12**)	1.1 µM	5	
No-PDE I206W	Bis-p-nitrophenyl Phosphate (**1**)	10.4 nM	2.4	8
No-PDE I206W	Bis-phenyl Phosphate (**2**)	433 nM	2.72	64
No-PDE I206W	4-Nitrophenyl Phosphate (**11**)	104 nM	5.2	16
No-PDE I206W	Phenyl Phosphate (**12**)	4.3 µM	4	188
No-PDE I206A	Bis-p-nitrophenyl Phosphate (**1**)	4.5 nM	2.4	8
No-PDE I206A	Bis-phenyl Phosphate (**2**)	94 nM	3.4	64
No-PDE I206A	4-Nitrophenyl Phosphate (**11**)	23 nM	5.2	16
No-PDE I206A	Phenyl Phosphate (**12**)	942 nM	4	188
No-PDE S209Q	Bis-p-nitrophenyl Phosphate (**1**)	7.3 nM	2.4	8
No-PDE S209Q	Bis-phenyl Phosphate (**2**)	30 nM	3.4	64
No-PDE S209Q	4-Nitrophenyl Phosphate (**11**)	73 nM	5.2	16
No-PDE S209Q	Phenyl Phosphate (**12**)	3.0 µM	5	188
No-PDE S209A	Bis-p-nitrophenyl Phosphate (**1**)	10.4 nM	2.4	8
No-PDE S209A	Bis-phenyl Phosphate (**2**)	22 nM	3.4	64
No-PDE S209A	4-Nitrophenyl Phosphate (**11**)	52 nM	5.2	16
No-PDE S209A	Phenyl Phosphate (**12**)	2.2 µM	5	188
No-PDE F239L	Bis-p-nitrophenyl Phosphate (**1**)	35 nM	2.4	8
No-PDE F239L	Bis-phenyl Phosphate (**2**)	29 nM	3.4	64
No-PDE F239L	4-Nitrophenyl Phosphate (**11**)	69 nM	5.2	16
No-PDE F239L	Phenyl Phosphate (**12**)	1441	5	188
No-PDE F239A	Bis-p-nitrophenyl Phosphate (**1**)	39 nM	2.4	8
No-PDE F239A	Bis-phenyl Phosphate (**2**)	164 nM	3.4	64
No-PDE F239A	4-Nitrophenyl Phosphate (**11**)	79 nM	5.2	16
No-PDE F239A	Phenyl Phosphate (**12**)	1.6 µM	5	188
No-PDE L263Q	Bis-p-nitrophenyl Phosphate (**1**)	53 nM	2.4	8
No-PDE L263Q	Bis-phenyl Phosphate (**2**)	44 nM	2.5	47
No-PDE L263Q	4-Nitrophenyl Phosphate (**11**)	533 nM	5.2	16
No-PDE L263Q	Phenyl Phosphate (**12**)	2.2 µM	5	188
No-PDE L263A	Bis-p-nitrophenyl Phosphate (**1**)	32 nM	2.4	8
No-PDE L263A	Bis-phenyl Phosphate (**2**)	132 nM	3.4	64
No-PDE L263A	4-Nitrophenyl Phosphate (**11**)	32 nM	5.2	16
No-PDE L263A	Phenyl Phosphate (**12**)	1.3 µM	5	188
No-PDE E268D	Bis-p-nitrophenyl Phosphate (**1**)	41 nM	2.4	8
No-PDE E268D	Bis-phenyl Phosphate (**2**)	169 nM	3.4	64
No-PDE E268D	4-Nitrophenyl Phosphate (**11**)	405 nM	5.2	16
No-PDE E268D	Phenyl Phosphate (**12**)	1.7 µM	4	188
No-PDE E268A	Bis-p-nitrophenyl Phosphate (**1**)	65 nM	2.54	8
No-PDE E268A	Bis-phenyl Phosphate (**2**)	269 nM	3.4	64
No-PDE E268A	4-Nitrophenyl Phosphate (**11**)	65 nM	5.32	17
No-PDE E268A	Phenyl Phosphate (**12**)	2.7 µM	4	94

[a]Titrations were done by serial dilutions consisting of 18 or 32 concentrations between high and low values given.

MATERIALS AND METHODS

In general lab supplies and chemicals were from Fisher Scientific. Pipette tips were from Rainin. Silica Gel (60 mesh) and reagents for synthesis of compounds were from Millipore Sigma. Solvents used were from Fisher Scientific or Pharmco. Restriction enzymes, DNA polymerase, and dNTPs were from New England Biolabs. pET vectors were from Millipore Sigma. DNA oligos were custom synthesized by Thermo Fisher. DNA sequences were verified by dideoxy sequencing at the Oklahoma Medical Research Foundation DNA Laboratory. NMR spectra were recorded on a Bruker 300 mHz Avance III spectrometer.

Chemicals tested. Diesters derived from flame retardants known or suspected to be carcinogenic (bis-2-chloroethyl phosphate (**3**), bis-1,3-dichloroisopropyl phosphate (**4**) and bis-2,3-dibromopropyl phosphate (**5**)), endocrine disruptors (bis-2-butoxyethyl phosphate (**6**)), and developmental toxins (diphenyl phosphate (**2**)) along with the model compound bis-*p*-nitrophenyl phosphate (**1**) were selected for study.[1-6] Additionally, the diesters from industrial compound tributyl phosphate and tricyclohexyl phosphate, which are used as solvents in the nuclear industry, along with diethyl (**8**) and dimethyl phosphate (**9**), which are derived from common insecticides, were tested.[7-9] To distinguish the diesterase activity from phosphatase activity the phosphomonoesters *p*-nitrophenyl phosphate (**11**) and phenyl phosphate (**12**) were utilized. The remaining monoesters discussed were derived *in-situ* from the enzymatic hydrolysis of the diesters.

Synthesis of compounds. Bis-*p*-nitrophenyl phosphate (**1**), diphenyl phosphate (**2**), dibutyl phosphate (**7**), *p*-nitrophenyl phosphate (**11**) and phenyl phosphate (**12**) were purchased from Millipore Sigma. Phosphochloridates of the remaining compounds were synthesized with modification of existing methods as described below followed by hydrolysis to the final compounds.[10] Compounds tested are shown in **Figure S1**. For all compounds purified by silica gel chromatography, the compound was loaded

on the column prepared in pure hexanes, washed with 100 mL hexanes and eluted with the hexanes/ethyl acetate mixtures given below.

Synthesis of bis-2-chloroethyl chlorophosphate. To 100 mL of diethyl ether, 1.8 mL of 2-chloroethanol (2.16 g, 26.9 mmol, 2 eq) was added with stirring and chilled to 0 °C in an ice bath. To this mixture 1.24 mL of trichlorophosphate (2.04 g, 13.45 mmol, 1 eq) and 3.75 mL triethyl amine (2.72 g, 2 eq) were added. The atmosphere was purged with nitrogen, and the reaction was allowed to come to room temperature and stirred overnight (~16 hr). Solid triethyl amine hydrochloride was filtered out and the solvent was removed under reduced pressure rotary evaporation to yield the crude product as a yellow oil. The crude product was purified by silica gel chromatography using 1:1 (hexanes: ethyl acetate) as the mobile phase. (Note: multiple spots could be visualized on TLC by incubating plates in an I_2 atmosphere followed by Hanessian's stain without heating) The purification yielded 2.04 g (63%) of bis-(2-chloroethyl) chlorophosphate as a yellow oil.

^{1}H NMR (300.13 MHz, $CDCl_3$) δ 4.577-4.382 (m, 4H), 3.78 (m, 4H). ^{31}P NMR (121.49 MHz, $CDCl_3$) δ 4.59 ppm.

Synthesis of bis-(2-chloroethyl) phosphate (3). Bis-(2-chloroethyl) chlorophosphate (2.04 g, 8.45 mmol) was added to 8.45 mL THF with stirring and cooled to 0 °C in an ice bath. To this mixture, 4.22 mL of H_2O was added followed by the addition of 4.22 mL of 2 M aqueous NaOH (1 eq) dropwise. The reaction was stirred for 1 hr at 0 °C. The reaction was diluted to 50 mL using 50 mM Hepes pH 8.0 and extracted with DCM (3 x 30 mL). The aqueous phase was acidified below pH 1 by dropwise addition of concentrated HCl and extracted with DCM (3 x 30 mL). The organic layer was dried over anhydrous $NaSO_4$. After removal of the solvent a small amount of product (140 mg) was recovered from the organic phase, but the majority remained in the aqueous phase. The water was removed via reduced pressure rotary evaporation and the resultant wet solid was extracted with diethyl ether yielding the desired product as

well as several contaminants as a pale-yellow oil after removal of solvent. The impure product was dissolved in 50 mL of 50 mM Hepes pH 8.0 and extracted with DCM (3 x 30 mL), acidified below pH 1 by addition of concentrated HCl, and reextracted with DCM (3 x 30 mL). The remaining aqueous layer was dried by reduced pressure rotary evaporation and the resulting wet solid was extracted with minimal DCM to yield 600 mg (40 %) pure product.

^{1}H NMR (300.13 MHz, $CDCl_3$) δ 4.99 (s, 1H), 4.34-4.28 (m, 4H), 3.74 (t, J= 5.80 Hz, 4H). ^{31}P NMR (121.49 MHz, $CDCl_3$) δ -0.66 ppm. ^{31}P NMR (121.49 MHz, D_2O/Hepes pH 8.0) δ -0.23 (qu, J= 6.68 Hz).

Synthesis of bis-1,3-dichloroisopropyl chlorophosphate. To 100 mL of diethyl ether, 2.55 mL of 1,3-dichloro-2-propanol (26.9 mmol, 3.47g, 2 eq) and 1.24 mL trichlorophosphate (2.04 g, 13.45 mmol, 1 eq) was added with stirring. The mixture was chilled to 0 °C, and 3.75 mL (2.72 g, 2 eq) of triethyl amine was added. The atmosphere was purged with nitrogen and the reaction was allowed to warm to room temperature. The reaction was stirred overnight (~16 hr). The reaction was filtered to remove solid triethylamine hydrochloride, and the solvent removed via reduced pressure rotary evaporation, yielding the crude product as a yellow oil. The product was further purified via silica gel chromatography using a 10% step gradient of hexanes and ethyl acetate (0-40% ethyl acetate) with 100 mL volumes for each step. The purified product contained only a single phosphorus species, but NMR indicated that there was a major (43%) alcohol side product. Approximately 900 mg (30 %) of the expected product was obtained and used without further purification.

^{1}H NMR (300.13 MHz, $CDCl_3$) δ 4.99-4.89 (m, 4H), 3.72 (d, J= 5.89 Hz, 4H). ^{31}P NMR (121.49 MHz, $CDCl_3$) δ 3.62 ppm (^{1}H coupled: t, J = 10.05Hz).

Synthesis of bis-1,3-dichloroisopropyl phosphate (4). The partially purified bis-(1,3-dichloroisopropyl) chlorophosphate (1.56 g (900 mg pure), 2.67 mmol, 1 eq) was added to 2.67 mL THF and cooled to 0 °C in an ice bath. 1.3 mL of H_2O was added with stirring and 1.3 mL of 2 M NaOH (1 eq) was added

dropwise to the mixture. The reaction was stirred on ice for 1.5 hr. The reaction was diluted to 50 mL with 50 mM Hepes (pH = 8.0) and extracted with DCM (3 x 30 mL). The aqueous layer was acidified to below pH 1 with concentrated HCl and reextracted with DCM (3 x 30 mL). The organic layer was dried over anhydrous Na_2SO_4 and the solvent removed via reduced pressure rotary evaporation yielding 609 mg (72 %) pure product.

^{1}H NMR (300.13 MHz, $CDCl_3$) δ 5.85 (s, 1H), 4.77-4.69 (m, 2H), 3.87 (d, J= 5.16 Hz, 8H). ^{31}P NMR (121.49 MHz, $CDCl_3$) δ -2.34 ppm. ^{31}P NMR (121.49 MHz, D_2O/Hepes pH 8.0) δ -2.28 (t, J= 8.77 Hz).

Synthesis of Bis-2,3-dibromopropyl chorophosphate. Trichlorophosphate (19.5 mmol, 3.0g, 1,8 mL, 1 eq) was mixed with 2,3-dibromo-1-propanol (39 mmol, 7.92 g, 3.7 mL, 2 eq) in 100 mL diethyl ether with stirring. The mixture cooled to 0 °C in an ice bath and 5.4 mL of triethyl amine (3.9 g, 2 eq) was added. The reaction was allowed to warm to room temperature (22 °C) and proceeded overnight (~16 hr). The reaction was filtered to remove solid and the solvent was removed under reduced pressure rotary evaporation. The crude product was then purified by silica gel column using a 3:1 mixture of hexane:ethylacetate, yielding 1.1 g (10 % yield) bis-2,3-dibromopropyl chlorophosphate as a yellow oil. Final product was approximately 75 % pure as judged by ^{31}P NMR and used without further purification. The three enantiomers were observed in a 1:1.94:0.72 ratio.

^{1}H NMR (300.13 MHz, $CDCl_3$) δ 4.73-4.55 (m, 4H), 4.24-4.32 (m, 2H) 3.91-3.73 (m, 4H). ^{31}P NMR (121.49 MHz, $CDCl_3$) δ 4.34 (s), 4.18 (s), 3.92 (s).

Synthesis of bis-2,3-dibromopropyl phosphate (5). Bis-2,3-dibromopropyl chlorophosphate (1.4 g, 2.7 mmol, 1 eq) was added to 2.7 mL THF and chilled on ice with stirring. 1.36 mL of H_2O and 1.36 mL of 2M NaOH (1 eq) were added dropwise, and the reaction was stirred for 3 hr on ice. The reaction was diluted with 50 mL of 50 mM Hepes pH 8.0 and extracted with DCM (3 x 30 mL). The aqueous layer was acidified by the addition of concentrated HCl until the pH was below 1 and extracted with DCM (3 x 30 mL). The

organic layer was dried over anhydrous Na_2SO_4 and the solvent removed under reduced pressure rotary evaporation. The resulting product yielded 716 mg of a yellow liquid (53 % yield). ^{31}P NMR determined the product was 87% pure, and it was used without further purification.

^{1}H NMR (300.13 MHz, $CDCl_3$) δ 4.58-4.41 (m, 4H), 4.40-4.28 (m, 2H) 3.94-3.74 (m, 4H). ^{31}P NMR (121.49 MHz, D_2O 50 mM Hepes pH 8.0) δ -0.84- -1.02 (qu, J = 5.54 Hz).

Synthesis of bis-2-butoxyethyl chlorophosphate. Trichlorophosphate (28 mmol, 4.28 g, 2.6 mL, 1.1 eq) was mixed with 2-butoxyethanol (52 mmol, 6.14 g, 6.82 mL, 2eq) in 100 mL of diethyl ether with stirring. The mixture was chilled in an ice bath, and 7.23 mL Triethyl amine (52 mmol, 5.25 g, 2 eq) was added. The environment was purged with nitrogen, and the reaction was allowed to warm to room temperature (22 °C) and stirred overnight (~16 hr). The solid was removed by filtration and the solvent was removed by reduced pressure rotary evaporation yielding a crude product as a yellow oil. Product was purified by silica gel chromatography using 3:1 (hexanes:ethylacetate) mixture to elute. After removing solvent, Bis-2-butoxyethyl chlorophosphate (5.87 g, 71 % yield) was recovered as yellow oil.

^{1}H NMR (300.13 MHz, $CDCl_3$) δ 4.37-4.31 (m, 4H), 3.72-3.69 (t, J = 4.68 Hz, 4H) 3.53-3.49 (t, J= 6.53 Hz, 4H) 1.66-1.54 (m, 4H), 1.43-1.28 (m, 4H), 0.97-0.92 (t, J = 7.29 Hz, 6H). ^{31}P NMR (^{1}H coupled) (121.49 MHz, $CDCl_3$) δ 5.51-5.22 (qu, J = 8.81 Hz).

Synthesis of bis-2-butoxyethyl phosphate (6). Bis-2-butoxyethyl chlorophosphate (2g, 6.3 mmol, 1eq) was dissolved in 6.3 mL THF and chilled in an ice bath. Water (3.15 mL) was added followed by the addition of 3.15 mL of 2 M NaOH (1 eq). Reaction was stirred on ice for 4 hr. Reaction was diluted with 50 mL Hepes buffer (50 mM, pH = 8.0) and extracted against DCM (3 X 30 mL). The aqueous phase was acidified with concentrated HCl until the pH was below 1 and then extracted with dichloromethane (3 X 30 mL). The organic layer was dried over Na_2SO_4 and the solvent removed by reduced pressure rotary evaporation to yield the product as a yellow oil. The final product contained a 13 % contaminant;

however, it was determined to be non-hydrolyzable and hence did not interfere with enzymatic assays. The reaction produced 803 mg of product with a yield of 43 % after purification.

^{1}H NMR (300.13 MHz, $CDCl_3$) δ 4.21-4.16 (m, 4H), 3.68-3.65 (m, 4H) 3.52-3.48 (t, J= 6.63 Hz, 4H) 1.63-1.53 (m, 4H), 1.45-1.35 (m, 4H), 0.96-0.91 (t, J = 7.32 Hz, 6H). ^{31}P NMR (^{1}H coupled) (121.49 MHz, D2O/Hepes pH = 8.0) δ 0.64-0.44 (qu, J = 6.05 Hz).

Synthesis of diethyl chlorophosphate. Ethanol (13 mmol,0.76 mL, 0.6 g, 2 eq) and trichlorophosphate (6.5 mmol, 1.0g, 600 µL, 1 eq) was mixed in 50 mL diethyl ether with stirring. The mixture cooled to 0 °C in an ice bath and 1.8 mL of triethyl amine (1.3g, 2 eq) was added. The solution was allowed to warm to room temperature and stirred for 2 hr. The reaction was filtered to remove solid and the solvent was removed under reduced pressure rotary evaporation to yield a light-yellow oil as the crude product. The crude mixture was purified over silica gel column using a 2:1 mixture of hexane:ethylacetate, yielding 357 mg (32%) diethyl chlorophosphate as a yellow oil.

^{1}H NMR (300.13 MHz, $CDCl_3$) δ 4.36-4.24 (m, 4H), 1.44 (t, J= 7.08 Hz, 6H) ^{31}P NMR (121.49 MHz, $CDCl_3$, ^{1}H coupled) δ 4.42 (qu, J = 9.00 Hz)

Synthesis of diethyl phosphate (8): Diethyl chlorophosphate (357 mg, 2.05 mmol, 1 eq) was dissolved in 2 mL THF and chilled to 0 °C on ice. To this solution, 1 mL of water and 1 mL of 2 M NaOH (1 eq) was added dropwise and the reaction stirred on ice for 1 hr. The reaction was diluted with to 30 mL with saturated sodium bicarbonate and extracted with dichloromethane (3 x 30 mL). The pH was brought below 1.0 by addition of concentrated HCl and the solution extracted with DCM (3 x 30 mL). The remaining aqueous solution was then dried under reduced pressure rotary evaporation to yield a white semi crystalline crude product. This product was washed with minimal DCM to extract the product. The solvent was removed yielding 125 mg (40% yield) of diethyl phosphate as a pale-yellow oil.

^{1}H NMR (300.13 MHz, $CDCl_3$) δ 6.19 (s, 1H), 4.13 (qu, J= 7.26 Hz, 4H), 1.37 (t, J= 7.02 Hz, 6H). ^{31}P NMR (121.49 MHz, $CDCl_3$) δ 1.25. ^{31}P NMR (121.49 MHz, D_2O/Hepes pH 8.0) δ 0.67 (qu, J= 7.19 Hz).

Synthesis of dimethyl chlorophosphate. Methanol (1.47 mL, 1.16g, 36.4 mmol, 2 eq) was added to 50 mL of diethyl ether and cooled to 0 °C in an ice bath. To this solution 1.7 mL of trichlorophosphate (1eq, 2.8 g) was added followed by 5 mL of triethyl amine (2 eq, 3.6 g). The solution was allowed to warm to room temperature and stirred for 16 hr. Reaction was filtered to remove solid triethylamine hydrochloride, and the solvent was removed under reduced pressure rotary evaporation to yield a yellow oil as the crude product. The product was purified using silica gel chromatography with hexanes:ethylacetate (1:1) as the mobile phase resulting in the recovery of 491 mg of pure dimethyl chlorophosphate (19 % yield).

^{1}H NMR (300.13 MHz, $CDCl_3$) δ 3.93 (d, J= 13.68 Hz, 6H), ^{31}P NMR (121.49 MHz, $CDCl_3$) δ 7.51(sept, J = 13.51 Hz).

Synthesis of dimethyl phosphate (9). Dimethyl chlorophosphate (491 mg, 3.41 mmol 1 eq) was dissolved in 3.4 mL of THF and chilled to 0 °C in an ice bath. To this solution 1.7 mL water and 1.7 mL of aqueous 2 M NaOH was added dropwise. The reaction was stirred on ice for 1 hr. The reaction was diluted to 50 mL with saturated sodium bicarbonate and extracted with DCM (3 x 30 mL). The aqueous layer was acidified with concentrated HCl until the pH was below 1.0 and then extracted with DCM (3 x 30 mL). The resulting aqueous layer was dried with reduced pressure rotary evaporation and the resulting solid extracted with minimal DCM to recover 126 mg of dimethyl phosphate (29 %) after solvent removal.

^{1}H NMR (300.13 MHz, $CDCl_3$) δ 3.79 (d, J= 11.19 Hz, 6H), 2.88 (s, 1H) ^{31}P NMR (121.49 MHz, $CDCl_3$) δ 2.93. ^{31}P NMR (121.49 MHz, D_2O/Hepes pH 8.0) δ 0.67 (sept, J= 11.21 Hz).

Synthesis of dicyclohexyl cholorophosphate. Cyclohexanol (1.3 g, 13 mmol, 2eq) was added to was added to 50 mL of diethyl ether and cooled to 0 °C in an ice bath. To this solution 0.6 mL of trichlorophosphate (1eq, 1 g) was added followed by 5 mL of triethyl amine (2 eq, 1.3 g). The solution was allowed to warm to room temperature and stirred for 16 hr. Reaction was filtered to remove solid triethylamine hydrochloride, and the solvent was removed under reduced pressure rotary evaporation to yield a yellow oil as the crude product. The product was purified using silica gel chromatography with hexanes:ethylacetate (5:1) as the mobile phase resulting in the recovery of 650 mg of pure dicyclohexyl chlorophosphate (36 % yield).

^{1}H NMR (300.13 MHz, $CDCl_3$) δ 4.64-4.53 (m, 2H), δ 2.08-1.93 (m, 4H) δ 1.8-1.72 (m, 4H) δ 1.70-1.47 (m, 6H) δ 1.461.20 (m, 6H)^{31}P NMR (121.49 MHz, $CDCl_3$) δ 2.32 (t, J = 9.61 Hz).

Synthesis of dicyclohexyl phosphate (10). Dicyclohexyl chlorophosphate (650 mg, 2.31 mmol 1 eq) was dissolved in 2.31 mL of THF and chilled to 0 °C in an ice bath. To this solution 1.56 mL water and 1.56 mL of aqueous 2 M NaOH was added dropwise. The reaction was stirred on ice for 1 hr. The reaction was diluted to 50 mL with saturated sodium bicarbonate and extracted with DCM (3 x 30 mL). The organic layer was dried and the solvent removed under reduced pressure rotary evaporation to yield 317 mg of dicyclohexyl phosphate (52% yield) as an oil.

^{1}H NMR (300.13 MHz, $CDCl_3$) δ 4.38-4.27 (m, 2H), δ 2.03-1.90 (m, 4H) δ 1.84-1.69 (m, 4H) δ 1.65-1.45 (m, 6H) δ 1.42-1.20 (m, 6H); ^{31}P NMR (121.49 MHz, H2O pH 8.0) δ -0.655 (t, J = 7.51 Hz).

Identification of *Sb*-PDE homologs. The sequence for *Sb*-PDE (NCBI ID: BAX25588) was submitted to the Enzyme Function Initiative Enzyme Similarity Tool (EFI-EST) to retrieve all sequences from Uniprot with a E-value of 10^{-20} or less.[11] As per the EFI protocol the retrieved sequences were then used in an all by all BLAST analysis to create a network of evolutionary relationships using an alignment score of 40 (~30% identity) as the cutoff. The created network was analyzed using Cytoscape and views generated with

the embedded j-files organic layout.[12] Close homologs of *Sb*-PDE were identified by clustering with an alignment score cut-off of 85 or greater.

Sequence alinements and substrate modeling. The protein sequences for *Sb*-PDE, *No*-PDE and PDB:3E38 excluding the export tags were aligned using the Clustal W algorithm in the SnapGene software from Dotmatics. The crystal structure of pdb:3E38 was analyzed and figures produced using Chimera from UCSF.[13] The substrate diphenyl phosphate was modeled in the active site using the Chimera build structure function to replace the cacodylate bond to the metal center in the published structure. The diphenyl phosphate was placed with the phosphate core in the same orientation as the original cacodylate with the two free oxygens bond to the α- and β- metals and the presumed leaving group oxygen within ligation distance to the γ-metal. The bond angels and rotation of the two phenyl ester groups were manually adjusted to minimize steric clash with the protein structure. The docked substrate was then subjected to two rounds of energy minimization using the default settings in Chimera, while holding the protein structure constant to identify the optimal binding orientation for the substrate.

Cloning of *No*-PDE and *Sb*-PDE. The gene for diesterase from Novosphingobium sp. EMRT-2 (*No*-PDE, NCBI ID: QCI9559) was obtained as the optimized synthetic gene sequence including N-terminal leader sequence inserted between the NdeI and XhoI sites of pET 28a from Twist Bioscience. Expression tests using bis-*p*-nitrophenyl phosphate (**1**) demonstrated clear diesterase activity which was lacking in an empty vector control. Despite *Sb*-PDE only differing in amino acid sequence from *No*-PDE by four amino acids, multiple attempts to clone a codon optimized version of *Sb*-PDE failed to yield any active protein. Given the close similarity to *No*-PDE the correct sequence for *Sb*-PDE was obtained by site directed mutagenesis of the *No*-PDE gene which yielded active *Sb*-PDE. Multiple attempts to clone a third homolog from *Sphingobium indicum* (NCBI ID: WP_006949123.1) failed to yield active protein for undetermined reasons.

Site Directed Mutagenesis. Additional variants were created using site directed mutagenesis. In brief, fully overlapping pairs of primers were designed with the minimal number of changes to introduce the desired change in amino acid. The mutagenic site was centered in the oligonucleotide with sufficient base pairs on either side to obtain a melting temperature of 78 °C according to the equation;

T_m = 81.5 + 0.41 x %GC – 675/n - % mismatch

where n is the number of base pairs in the oligonucleotide not including mismatched base pairs.

Oligonucleotides were used in PCR reactions with Phusion polymerase from New England Biolabs. Final PCR mix was 50 µL total volume with 1 µL Phusion polymerase, 1X Phusion buffer, 0.5 mM of each dNTP, 125 ng each of forward and reverse primers, and 0.5 µL of template DNA from a standard plasmid prep. Thermocycler program consisted of an initial 1 min @ 95 °C followed by 25 cycles of 45 sec @ 94 °C, 45 sec @ 65 °C, 3 min @ 72 °C, and a final 10 min @ 72 °C. For reactions that did not initially yield product the forward and reverse primers were used separately in 25 µL reactions as described above for 10 thermocycles and then the reactions were combined for an additional 15 cycles which yielded the desired products in all cases.

PCR products were digested with DpnI restriction enzyme for 2 hr in the PCR condition, and were then purified using the Monarch PCR & DNA Cleanup Kit from New England Biolabs. Products were eluted in 30 µL DNase free water (Fisher Scientific). BL21 (DE3) competent cells were then transformed with 1 µL of the cleaned products using electroporation.

Protein expression and purification. Freshly transformed colonies were inoculated into 1 mL overnight cultures of LB containing 50 µg/mL kanamycin. The following morning 1 L cultures of LB were inoculated with the overnight cultures and grown at 37 °C until they reached an OD_{600} of 0.7. Temperature was reduced to 30 °C and protein expression was induced by addition of 0.1 mM IPTG. Expression continued

for 16 hr at 30 °C before cells were harvested by centrifugation (10 min @ 8k RPM). Cell pellets were stored at -80 °C prior to use.

Cell pellets from 2L of culture were resuspended in 100 mL 50 mM Hepes (pH = 8.0) and sonicated a total of 10 min (5 cycles of 2 min with 10 s on 15 s off with 5 min rest between cycles) using a Fisher Scientific Sonic Dismembrator 500. Cell debris was removed via centrifugation (10 min @ 10k RPM). The supernatant was brought to 0.2 % protamine sulfate by the dropwise addition of a 2.2% stock solution in 50 mM Hepes (pH = 8.0) and stirred on ice for 20 min. The precipitated nucleotide component was removed via centrifugation (10 min @ 10k RPM). The resulting supernatant was brought to 35 % (19.4 g/100 mL) saturation with ammonium sulfate and stirred on ice for 20 min. Precipitated protein was removed via centrifugation (10 min @ 10k RPM). The resultant supernatant was brought to 80% saturation (additional 29.1 g/100 mL) with ammonium sulfate to precipitate *No*-PDE. The protein was recovered via centrifugation (10 min @ 10k RPM). The pellet was resuspended in 4 mL 50 mL Hepes (pH 8.0) and loaded on a Cytiva HiPrep 26/600 Sephacryl 200 High Resolution size exclusion column equilibrated in the same buffer and attached to a Akta Prime FPLC system with a 1.3 mL/min flow rate. Fractions with activity were pooled and run over a gravity fed Cellulose DEAE resin in 50 mM Hepes (pH 8.0). Neither *No*-PDE nor *Sb*-PDE bind this resin, so flow through fractions were collected and purity checked via SDS-PAGE (Any Kd Stain Free TXG gels from BioRad). Pure fractions were pooled and the protein concentrated to greater than 2 mg/mL using an Amicon Ultra 15 (Ultracel 10K membrane) centrifugal ultra-filtration device. Aliquots of protein were flash frozen in liquid nitrogen and stored at -80 °C prior to use. Submission of the sequences to the SignalP 5.0 server resulted in a 99% prediction of a Sec/SPI cleavage site between residues 28 and 29.[14] The molecular weight of the intact *No*-PDE is 51.0 kDa, while the N-terminal cleavage results in a molecular weight of 45.8 kDa. SDS-PAGE analysis of *No*-PDE and *Sb*-PDE demonstrates that they consistently run ahead of a 50 kDa marker demonstrating that the signal peptide is efficiently processed in *E. coli*.

Metal dependence and analysis. *No*-PDE was tested for metal dependence as purified by supplementing assay with Zn, Co, Ni, or Mn. Assays were 1 mL total volume and contained 1 mM bis-p-nitrophenyl phosphate, 50 mM Ches pH 9 and the appropriate metal at 0 mM, 0.1 mM, or 1 mM. None of the metals resulted in a significant increase in activity indicating that the enzymes as purified were fully constituted. Incubating the enzyme with up to 40 mM EDTA or 1 mM 1,10-phenanthroline failed to inactivate the enzyme. Zinc content in the as purified samples was quantified by atomic absorption spectroscopy using a Persee AA990 flame spectrometer equipped with a nebulizer. Proteins samples were freed of exogenous metals by passing through Micro Bio-Spin P-30 desalting columns (BioRad) preequilibrated in 50 mM Hepes (pH -8.0). Enzymes samples (~100-300 µL) were digested by addition of 30 µL concentrated nitric acid. Following digestion samples were diluted to 5 µM protein concentration by addition of H_2O and passed through a 0.45 µM syringe filter. The spectrometer was calibrated with stock solutions of $ZnCl_2$ from 0 to 20 µM and samples were read 5 times each. Atomic Absorption Spectroscopy verified the presence of a trinuclear zinc site for both enzymes with *No*-PDE giving a ratio of 2.7 Zn/enzyme and *Sb*-PDE giving 2.9 Zn/enzyme. Metal analysis of all subsequent variants found > 2.3 Zn/enzyme.

NMR analysis of activity. For each substrate, 1 µL of substrate was dissolved in 50 µL of methanol and added to a 1 mL total volume reaction containing 50 mM Hepes pH 8.5, 20 % D_2O and 5 % MeOH. *No*-PDE or *Sb*-PDE (1 µM) was added and the reaction transferred to an NMR tube. The ^{31}P NMR spectra was recorded periodically over 72 hr for each sample as well as a matched no enzyme control. Product of diesterase and monoesterase reactions were identified by addition of phosphate in the case of aromatic substrates or by recording the proton coupled ^{31}P NMR spectra for alkyl diesters.

Spectrophotometric Kinetic Assays. The hydrolysis of bis-*p*-nitrophenyl phosphate (**1**) and *p*-nitrophenyl phosphate (**12**) were followed by the release of the *p*-nitrophenyl group at 400 nm (E_{400} = 17,000 $mM^{-1}cm^{-1}$) in a VersaMax 96-well plate reader (Molecular Devices). Reactions were 250 µL total volume with

50 mM Ches buffer (pH = 9.0) and 5 mM – 6 μM substrate. Diphenyl phosphate (**2**) and phenyl phosphate (**12**) were followed by the release of phenol at pH 9.0 (ΔE_{275} = 903 $M^{-1}cm^{-1}$) in a Beckman Coulter DU 640 spectrophotometer. Reactions were 1 mL total volume with 50 mM Ches pH 9.0 and 5.5 mM - 48 μM substrate. Stock solutions of substrate were made in water at 10 mM. Titrations were carried out by serial dilution of the stock solutions to yield 18 concentrations of compounds **2** and **12** or 32 concentrations for compounds **1** and **11** between the low and high concentrations listed in **Tables S2** and **S3**. All reactions were conducted at 22 °C and initiated by addition of appropriately diluted enzyme. Data were fit to Michalis Menten kinetics. For variants that failed to display sufficient saturation, the initial linear portion of the rate (v_o/[E_t]) vs [substrate] plot was fit to a straight line the slope of which yields the k_{cat}/K_m value for the enzyme at low concentration of substrate.

^{31}P NMR Kinetic Assays. The hydrolysis of bis-2-chloroethyl phosphate (**3**), bis-1,3-dichloroisopropyl phosphate (**4**), bis-2,3-dibromopropyl phosphate (**5**), bis-2-butoxyethyl-phosphate (**6**), dibutyl phosphate (**7**), and dicyclohexyl phosphate (**10**) were followed by ^{31}P NMR under pseudo first order conditions. Substrates were prepared as 40 mM stocks in methanol. Assays were 1 mL total volume with 5% methanol, 50 mM Hepes pH 8.0, 10 % D_2O, and 2 mM substrate. Reactions were initiated by addition of enzyme sufficient to hydrolyze approximately 50% of substrate in a 2 hr period. The ^{31}P NMR spectra was recorded every 15 min (120 scans, aqu = 3.5 sec, d1 = 4 sec) over 12 hrs. Reactions were conducted at 22 °C. Each enzyme was tested a minimum of twice with each substrate. The initial test determined the appropriate enzyme to yield 50 % hydrolysis, and then the full hydrolysis curve was recorded over 12 hr. The total phosphorus signal for each spectrum was integrated and used to calculate the fraction hydrolyzed for each time point. Plotting the fraction hydrolyzed as a function of time yielded first exponential curves that were fit to equation 1 to yield the exponential rate (k_{obs}) as previously described.[15]

$$F = a(1 - e^{-k_{obs}t}) \quad (1)$$

$$k_{cat}/K_m = \frac{k_{obs}}{[E]} \quad (2)$$

F is the fraction hydrolyzed, a is the magnitude of the exponential phase and t is time. The k_{cat}/K_m of the enzyme is calculated by dividing the exponential rate by the concentration of enzyme [E] in the reaction, as shown in equation 2. In some cases, the reactions demonstrated an initial linear phase followed by the expected first order exponential. In those cases, the data was reprocessed to include only the exponential phase.

AUTHOR INFORMATION

Corresponding Author

Andrew N. Bigley – Department of Chemistry and Physics, Southwestern Oklahoma State University, Weatherford, Oklahoma, 73093, United States

orcid.org/0000-0002-9612-1549; Email: andrew.bigley@swosu.edu

Authors

Preston Garner – Department of Chemistry and Physics, Southwestern Oklahoma State University, Weatherford, Oklahoma, 73093, United States

References

[1] Toxicology and carcinogenesis studies of 2-chloroethanol (ethylene chlorohydrin) (CAS No. 107-07-03) F344/N Rats and Swiss CD-1 Mice. (1985), National Toxicology Program, US Department of Health and Human Serivices National Institutes of Health NC, USA.

[2] 1.3-Dichloro-2-propanol (CAS No. 96-23-1) review of toxicological literature. (2005) In *US Department of Health and Human Serivices* (Program, N. T., Ed.).

[3] Isales, G. M., Hipszer, R. A., Raftery, T. D., Chen, A., Stapleton, H. M., and Volz, D. C. (2015) Triphenyl phosphate-induced developmental toxicity in zebrafish: potential role of the retinoic acid receptor, *Aquat Toxicol 161*, 221-230.

[4] Kwon, B., Shin, H., Moon, H. B., Ji, K., and Kim, K. T. (2016) Effects of tris(2-butoxyethyl) phosphate exposure on endocrine systems and reproduction of zebrafish (Danio rerio), *Environ Pollut 214*, 568-574.

[5] Organization, W. H. (1998) *Flame retardants: tris (chloropropyl) phosphate and tris (2-chloroethyl) phosphate*, World Health Organization.
[6] Prival, M. J., McCoy, E. C., Gutter, B., and Rosendranz, H. S. (1977) Tris(2,3-dibromopropyl) phosphate: mutagenicity of a widely used flame retardant, *Science 195*, 76-78.
[7] ORGANOPHOSPHORUS INSECTICIDES: A GENERAL INTRODUCTION. (1986), Geneva.
[8] Brahmmananda Rao, C. V. S., Suresh, A., Srinivasan, T. G., and Vasudeva Rao, P. R. (2003) Tricyclohexylphosphate—A Unique Member in the Neutral Organophosphate Family, *Solvent Extraction and Ion Exchange 21*, 221-238.
[9] Schulz, W. W., Navratil, J.D. (1984) *Science and technology of tributyl phosphate Vol I: Synthesis, properties, reactions and analysis.* , CRC Press., United States: .
[10] Santschi, N., Geissbuhler, P., and Togni, A. (2012) Reactivity of an electrophilic hypervalent iodine trifluoromethylation reagent with hydrogen phosphates-A mechanistic study, *J Fluorine Chem 135*, 83-86.
[11] Gerlt, J. A. (2017) Genomic Enzymology: Web Tools for Leveraging Protein Family Sequence-Function Space and Genome Context to Discover Novel Functions, *Biochemistry 56*, 4293-4308.
[12] Shannon, P., Markiel, A., Ozier, O., Baliga, N. S., Wang, J. T., Ramage, D., Amin, N., Schwikowski, B., and Ideker, T. (2003) Cytoscape: A Software Environment for Integrated Models of Biomolecular Interaction Networks, *Genome Research 13*, 2498-2504.
[13] Pettersen, E. F., Goddard, T. D., Huang, C. C., Couch, G. S., Greenblatt, D. M., Meng, E. C., and Ferrin, T. E. (2004) UCSF Chimera--a visualization system for exploratory research and analysis, *J. Comput. Chem. 25*, 1605-1612.
[14] Almagro Armenteros, J. J., Tsirigos, K. D., Sønderby, C. K., Petersen, T. N., Winther, O., Brunak, S., Von Heijne, G., and Nielsen, H. (2019) SignalP 5.0 improves signal peptide predictions using deep neural networks, *Nature Biotechnology 37*, 420-423.
[15] Bigley, A. N., Desormeaux, E., Xiang, D. F., Bae, S. Y., Harvey, S. P., and Raushel, F. M. (2019) Overcoming the Challenges of Enzyme Evolution To Adapt Phosphotriesterase for V-Agent Decontamination, *Biochemistry 58*, 2039-2053.
